# Identification of Odor-Processing Genes in the Emerald Ash Borer, *Agrilus planipennis*

**DOI:** 10.1371/journal.pone.0056555

**Published:** 2013-02-12

**Authors:** Praveen Mamidala, Asela J. Wijeratne, Saranga Wijeratne, Therese Poland, Sohail S. Qazi, Daniel Doucet, Michel Cusson, Catherine Beliveau, Omprakash Mittapalli

**Affiliations:** 1 Department of Entomology, The Ohio State University, Ohio Agricultural and Research Development Center, Wooster, Ohio, United States of America; 2 Department of Molecular and Cellular Imaging Center, The Ohio State University, Ohio Agricultural and Research Development Center, Wooster, Ohio, United States of America; 3 USDA Forest Service, Northern Research Station, Michigan State University, East Lansing, Michigan, United States of America; 4 Natural Resources Canada, Sault Ste. Marie, Ontario, Canada; 5 Natural Resources Canada, Québec, Canada; AgroParisTech, France

## Abstract

**Background:**

Insects rely on olfaction to locate food, mates, and suitable oviposition sites for successful completion of their life cycle. *Agrilus planipennis* Fairmaire (emerald ash borer) is a serious invasive insect pest that has killed tens of millions of North American ash (*Fraxinus* spp) trees and threatens the very existence of the genus *Fraxinus*. Adult *A. planipennis* are attracted to host volatiles and conspecifics; however, to date no molecular knowledge exists on olfaction in *A. planipennis*. Hence, we undertook an antennae-specific transcriptomic study to identify the repertoire of odor processing genes involved in *A. planipennis* olfaction.

**Methodology and Principal Findings:**

We acquired 139,085 Roche/454 GS FLX transcriptomic reads that were assembled into 30,615 high quality expressed sequence tags (ESTs), including 3,249 isotigs and 27,366 non-isotigs (contigs and singletons). Intriguingly, the majority of the *A. planipennis* antennal transcripts (59.72%) did not show similarity with sequences deposited in the non-redundant database of GenBank, potentially representing novel genes. Functional annotation and KEGG analysis revealed pathways associated with signaling and detoxification. Several odor processing genes (9 odorant binding proteins, 2 odorant receptors, 1 sensory neuron membrane protein and 134 odorant/xenobiotic degradation enzymes, including cytochrome P450s, glutathione-*S*-transferases; esterases, etc.) putatively involved in olfaction processes were identified. Quantitative PCR of candidate genes in male and female *A. planipennis* in different developmental stages revealed developmental- and sex-biased expression patterns.

**Conclusions and Significance:**

The antennal ESTs derived from *A. planipennis* constitute a rich molecular resource for the identification of genes potentially involved in the olfaction process of *A. planipennis*. These findings should help in understanding the processing of antennally-active compounds (e.g. 7-epi-sesquithujene) previously identified in this serious invasive pest.

## Introduction

Olfaction is the primary sensory perception modality in insects, guiding them to locate food (host cues), conspecifics (mating), suitable oviposition sites (access to nutritious and digestible food for off-spring), and to detect predators and toxic compounds [1]–[2]. Antennae are the principal biosensors for insect olfaction, where odorant messages (host plant volatiles, pheromones and predator odors) are translated into physiological signals (chemical and electric) that ultimately affect the insect's behavior [3]. Insect antennae are mobile, segmented and paired appendages with a basal scape, distal pedicel and flagellomeres that house sensilla or sensory hairs.

The majority of the insect sensilla contain 1–4 olfactory receptor neurons (ORNs) surrounded by accessory cells [4]. The ORNs act as biological transducers that convert ecologically relevant volatile chemicals into a sensory input. A number of perireceptor proteins and surface receptors have been identified as having roles in olfaction, depending on the odor context and ORNs involved. These include the odorant binding proteins (OBPs), odorant receptors (ORs), the sensory neuron membrane protein 1 (Snmp1), chemosensory proteins (CSPs), gustatory receptors (GRs) and ionotropic receptors (IRs).

OBPs are small, hydrophilic proteins that are secreted by the accessory cells and accumulate in the sensillum lymph. The function of OBPs in coleopteran insect communication is starting to be unraveled, with several OBPs having been isolated in moths, dipterans and hemipterans and displaying affinities to host plant volatile compounds or pheromones [5]–[7]. As their name implies, OBPs uptake volatile odors that enter the sensillum pore, bind physiologically relevant molecules and transport their cargo to the surface of ORNs [8]. OBPs serve as the liaison between the external environment and the odorant receptors (ORs) located on ORNs dendrites [8]. CSPs, like OBPs, are small molecular weight soluble proteins, some of which are expressed at high levels in the sensillum lymph and in other non-olfactory tissues. Though the role of CSPs in mediating chemoreception is unclear, their tissue-specific expression, ligand binding kinetics, and their binding affinity towards pheromones supports their putative role in insect olfaction [9].

Insect ORs have seven transmembrane domains with inverted membrane topology, a unique pattern found in insects compared with that of higher animals [10]. The OR family is extremely diverse, with the beetle *Tribolium castaneum* encoding up to 300 different OR genes [11], [12]. Studies in *Drosophila* have revealed the requirement of two ORs to transduce odor-evoked signals: an olfactory receptor coreceptor (Orco, previously known as OR83b [13], and a specific OR, which varies according to ORN type [14]). Orco is widely conserved across insects and is required for the trafficking and functioning of co-expressed ORs [15]. Snmp1 is a homolog of the mammalian CD36 and has been shown to be required for pheromone-evoked signaling in *Drosophila* [16], [17]. Snmp1 appears to play a role that is specific to pheromone perception, as it is dispensable in general odorant-evoked signaling [18].

Finally, the transmembrane IRs have been recently identified as receptors in odor-mediated signaling in *Drosophila*. Work in this insect model system by Benton et al. 2009 [19] showed that several IRs are expressed at the ciliated endings of some antennal receptor neurons. Furthermore, the ectopic expression of some IRs in specific sensilla triggers novel odor-evoked responses, implying a functional connection between IRs and odor perception.

Invasive insect pests represent a category of animals that have been successful in invading unoccupied ecological niches worldwide. The emerald ash borer (*Agrilus planipennis* Fairmaire) represents one such species at the present time. The accidental introduction of *A. planipennis* into North America in 2002 has so far resulted in the mortality of tens of millions of North American ash trees (*Fraxinus* spp.) and its spread continues, making the entire ash resource highly vulnerable to its attack [20]–[23]. Despite attaining “high alert” pest status, and considerable effort to develop traps and lures for early detection and implementation of management tactics that could help contain the spread of *A. planipennis*, to date little information exists on how this insect pest may perceive host volatiles and mate cues. Adult *A. planipennis* are attracted to host volatiles [24], [25] and to conspecifics [26]–[28], but discrepancies have been observed between the activity of certain volatiles, as measured by electroantennography or behavioral tests e.g. the bark volatile 7-epi-sesquithujene [29]. Identifying the antennal pathways that participate in volatile compound binding, transport and olfactory neuron response may help us understand why certain compounds elicit, or fail to elicit, behavioral activity in a laboratory or field setting.

With the advent of next generation sequencing methods (viz., 454 pyrosequencing and Illumina platforms), considerable progress in insect genomics has been achieved in the recent past [30]–[35]. For example, our recent study on tissue-specific (midgut and fat body) transcriptomics on *A. planipennis* using 454 pyrosequencing revealed a plethora of candidate genes involved in detoxification and provided insights into transcriptionally driven physiological adjustments [30]. For the present study, we applied 454 pyrosequencing to decipher the antennal transcriptome of *A. planipennis.* This effort should allow us to identify genes of *A. planipennis* that are potentially involved in recognizing host and mate cues leading to an increased understanding of *A. planipennis* olfaction.

## Results and Discussion

### Transcriptome assembly

**Figure 1:**
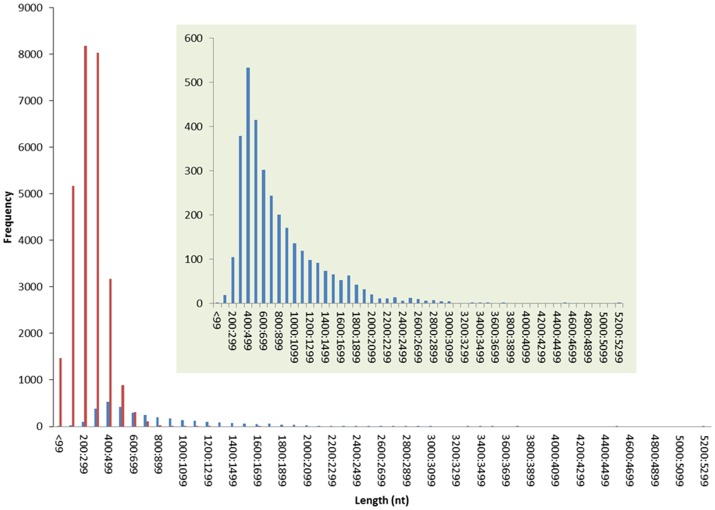
Summary of *Agrilus planipennis* antennal transcriptome. The isotigs and non-isotigs are represented by blue and red bars, respectively.

Sequencing of the *A. planipennis* antennal transcriptome resulted in a total of 37,399,265 bases and 139,085 reads. Contig assembly using Newbler and CAP3 assemblers generated a total of 30,615 high quality expressed sequence tags (ESTs). These ESTs included 3,249 isotigs and 27,366 non-isotigs (terms explained in Material and Methods), which were used for further analyses (Figure 1). The isotigs sequences ranged from 84 bp to 5,289 bp, with an average length of 828 bp and total length of 2,692,017 bp; whereas the non-isotigs ranged from 50 bp to 1,691 bp with an average length of 289 bp and total length of 7,929,112 (Table S1).

### Comparative genomics

**Figure 2:**
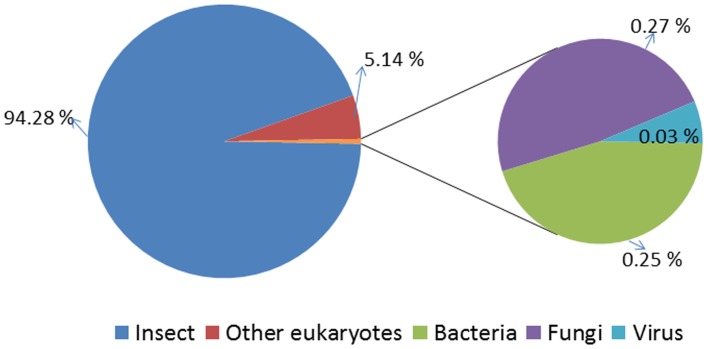
A pie chart showing species distribution of the top BLAST hits of *Agrilus planipennis* antennal transcripts.

**Figure 3:**
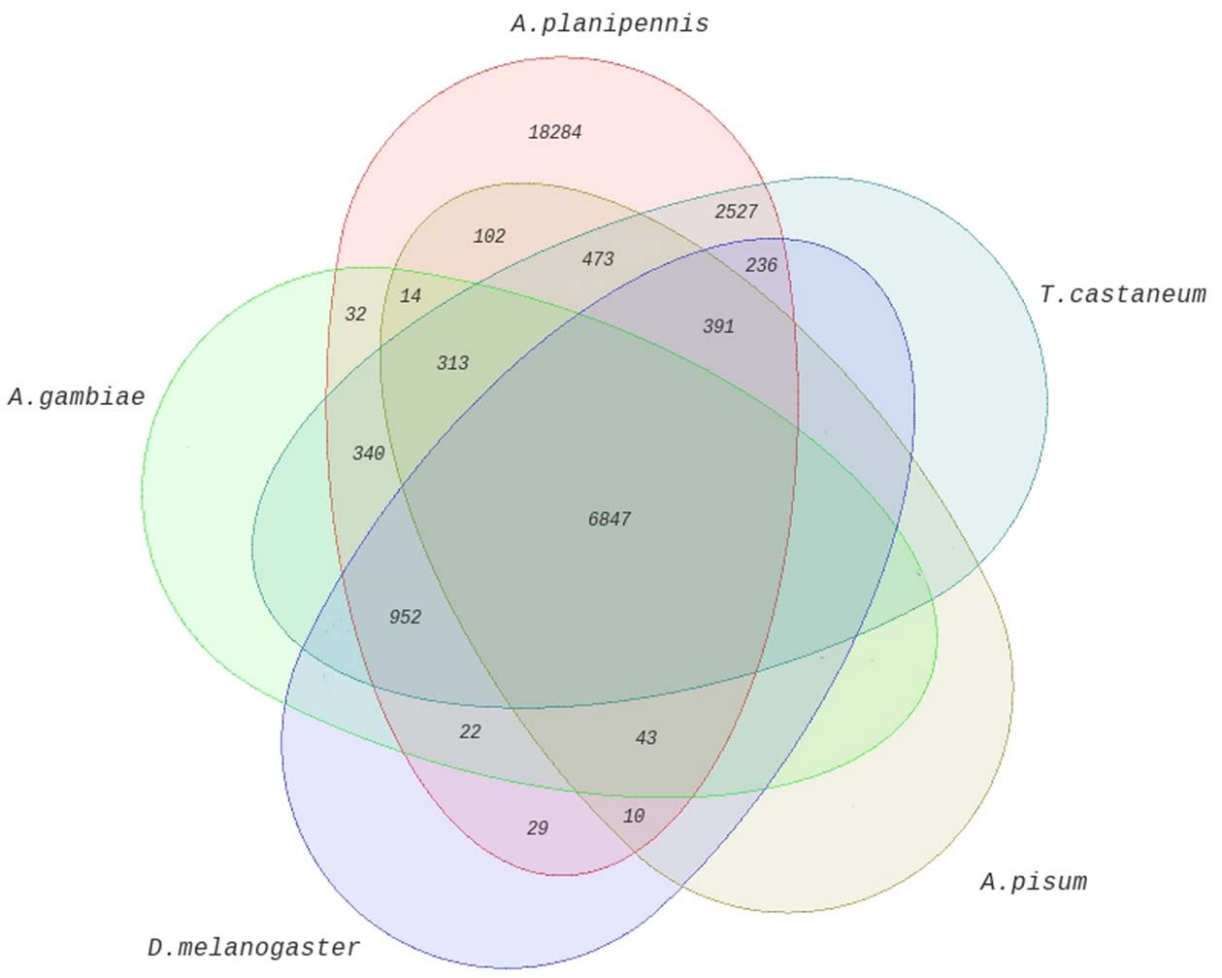
Comparative genomic analysis of *Agrilus planipennis* antennal transcripts. The 5-way Venn diagram shows the number of *A. planipennis* antennal transcripts shared with the four genomes of model insects (*Acyrthosiphon pisum, Anopheles gambiae, Drosophila melanogaster* and *Tribolium castaneum*).

Among the *A. planipennis* antennal ESTs obtained (30,615), a majority (18,284 sequences, 59.72%) (Table S2) did not show any similarity with proteins deposited in the non-redundant database of GenBank, potentially representing novel genes, as observed with the *Manduca sexta* antennal transcriptome [35]. The top BLAST hits of the *A. planipennis* ESTs showed high similarity with proteins from insects (94.28%) and other eukaryotes (5.14%), followed by proteins from bacteria (0.25%), fungi (0.27%) and viruses (0.03%) (Figure 2, Table S3). We then compared the *A. planipennis* antennal transcripts with the genomes of model insects (*Acyrthosiphon pisum* Harris, *Anopheles gambiae* Giles, *Drosophila melanogaster* Meigen, *Tribolium castaneum* Hebst); *A. planipennis* proteins revealed high sequence similarity (39.45%) with those of *T. castaneum*, the only beetle genome sequence available to-date (Figure 3) [36]. An approximately equal percentage of sequences showed similarity (27.96% *A. gambiae*, 27.86% *D. melanogaster* and 26.76% *A. pisum*) with the other three species compared (Figure 3). We also compared the current antennal ESTs with our previous *A. planipennis* midgut and fat body transcriptomic databases to examine tissue-specificity among the ESTs. This comparison revealed a higher percentage of transcripts similar to midgut transcripts (37.16%) than to fat body transcripts (23.75%) while 39.1% of transcripts were antennae-specific (Figure S1; Table S4). Results of this tissue-specific comparison of *A. planipennis* antennal sequences are in agreement with findings reported for the *M. sexta* antennal transcriptome [35].

### Gene ontology, Protein domains and KEGG analysis

**Figure 4:**
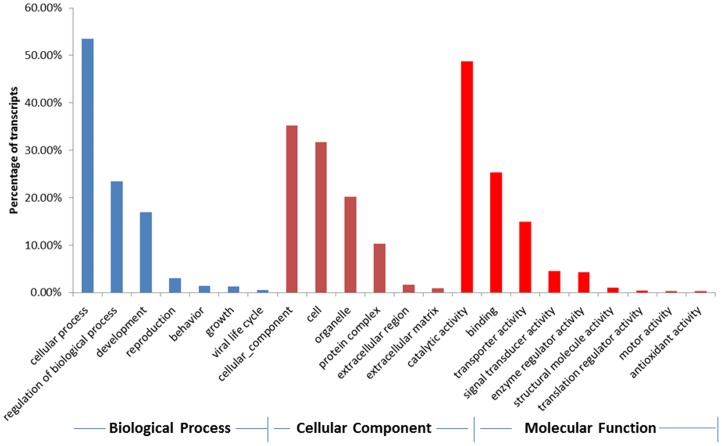
Gene Ontology (GO) analysis of *Agrilus planipennis* antennal transcripts. GO terms assigned to biological process (blue), cellular component (green), and molecular function (red).

Gene ontology (GO) assignments and Kyoto Encyclopedia of Genes and Genomes (KEGG) classifications were applied to the predicted antennal proteins. From the *A. planipennis* antennal ESTs obtained, a total of 5,486 antennal ESTs were assigned to various GO terms (3,350 Biological Process, 1,177 Cellular Component and 959 Molecular Function; Table S5). The major GO terms associated with Molecular Function were catalytic (48.80%), binding (25.34%), and transporter activity (14.91%), which potentially reflects the metabolic nature of the antennal tissue (Figure 4). The majority of the Biological Process and Cellular Component terms were associated with cellular process (53.46%) and cellular components (36.24%). Similar GO categories were also obtained for the midgut and fat body transcriptome of *A. planipennis* and antennal transcriptome of *M. sexta* [30], [35].

The Pfam domain search yielded 6,524 domains for 3826 transcripts (Table S6). Among the top 20 domains, immunoglobulin domains were the most abundant (314). Immunoglobulin's (Ig) are the largest category of cell surface proteins in insects, which assist in neuronal guidance and play important roles in insect immunity [37], [38]. A prominent example of the importance of such domain in insects can be found in the *Drosophila* Ig-containing receptor Dscam (Down syndrome cell adhesion molecule). Dscam can be spliced into 18,000 isoforms that direct neurite self-avoidance, but that are also involved in immune recognition [38], [39]. Next to immunoglobulin domains, we found a high number C_2_H_2_ Zinc finger proteins (ZFPs, 305) and WD40/YVTN (298) domains. While ZFPs represent the largest group of DNA/RNA binding proteins in eukaryotes, the WD40 repeats play a vital role in RNA processing, signal transduction and cytoskeleton assembly [40], [41]. Recent studies using RNA interference (RNA*i*) to reveal the function of the *abrupt* gene coding for ZFP in *T. castaneum* resulted in fusion of adjacent articles throughout the antenna [42]. We also identified Armadillo domains (201) in the current database which are thought to be involved in signal transduction and embryonic development [43]. The abundance of Major Facilitator Superfamily (MFS) domains (123) in the *A. planipennis* antennal transcriptome is in agreement with recent RNA-Seq studies on chemosensory tissues of *A. gambiae* [34]. The high occurrence of NAD(P) binding domain (89) might be involved in the biotransformation of odorant/toxic compounds [44]. Lastly, we found a high number of tetratricopeptides (TPP, 54) and Ankyrin repeat-containing domains (47) which are thought to be involved in several protein-protein interactions [45]–[48].

The KEGG analysis for the assigned sequences (2,270) resulted in 61 metabolic pathways with predominant sequences involved in purine metabolism (543), thiamine metabolism (435) and secondary metabolites (70) (Table S7). Similar trends in the KEGG assignment was found for midgut and fat body transcriptomes of *A. planipennis* [30].

### Genes of interest

We focused on gene families associated with odor processing and odor/xenobiotic degradation (Table 1). The retrieved number of odor reception ESTs (9 OBPs; 2 ORs; 1Snmp; 6 IRs; 6 Ionotropic Glutamate Receptors, IGluRs; 2 GRs; and 4 CSPs) in the derived *A. planipennis* antennal transcriptome reflects the depth of our sequencing, using one-quarter picotitre plate. Future genomic studies may provide the actual/entire complement set of genes involved in *A. planipennis* olfaction. The recent genome projects of the pea aphid (*A. pisum*) and body louse (*Pediculus humanus*) revealed a comparatively lower number of OBPs (15 and 5) and ORs (79 and 10) than in *T. castaneum* [36], [49]–[50]. These observations could reflect their mode of feeding and/or host specialization i.e., phloem feeding specialization (pea aphid) or obligate parasitism (body louse). On the other hand, *T. castaneum* has a large repertoire of olfactory genes (265 ORs; 49 OBPs; 19 CSPs) [36], [51]. From these observations, it is evident that the number of odor processing genes is highly variable among insect species and that this genomic diversity is linked to specific life history features, reproduction and mode of survival [36], [49]–[52].

**Table 1:** Summary of candidate genes from the antennal transcriptome of *Agrilus planipennis*.

Candidate genes	# in occurrence
*Odor-reception*	
**Odor binding proteins** *	**09**
**Odorant receptors**	**02**
Ionotropic receptors	06
Ionotropic glutamate receptors	06
Gustatory receptors	02
Chemosensory proteins	04
**Sensory neuron membrane proteins**	**01**
*Odor/xenobiotic degradation*	
**Cytochrome P450s**	**83**
Glutathione *S-*transferases	13
Esterases	56
Aldehyde dehydrogenases	31
Epoxide hydrolases	09
Catalases	04
Superoxide dismutase	10
Glutathione peroxidase	05

* Category of the candidate genes assayed in this study (in bold).

**Figure 5:**
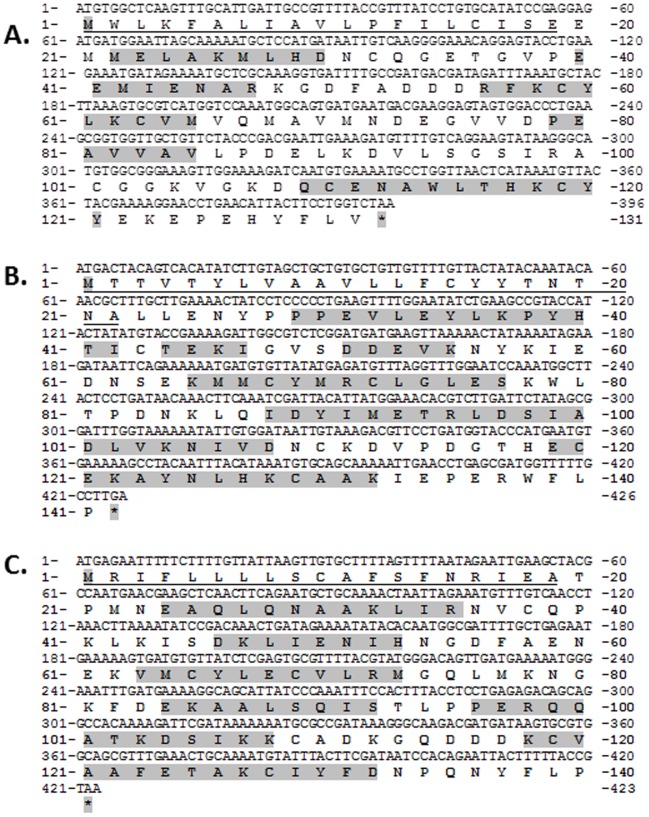
Predicted structural features of an odor binding proteins (OBPs) from *Agrilus planipennis* (A-C: *Ap*OBP1, *Ap*OBP3 and *Ap*OBP4) Nucleotide and deduced amino acid sequence of *Ap*OBP1. The start and stop codons are highlighted in dark gray. The putative signal peptide is underlined. The 5 predicted alpha helices are shaded in gray (the signal peptide sequence was removed before identifying alpha helical regions of the protein).

**Figure 6:**
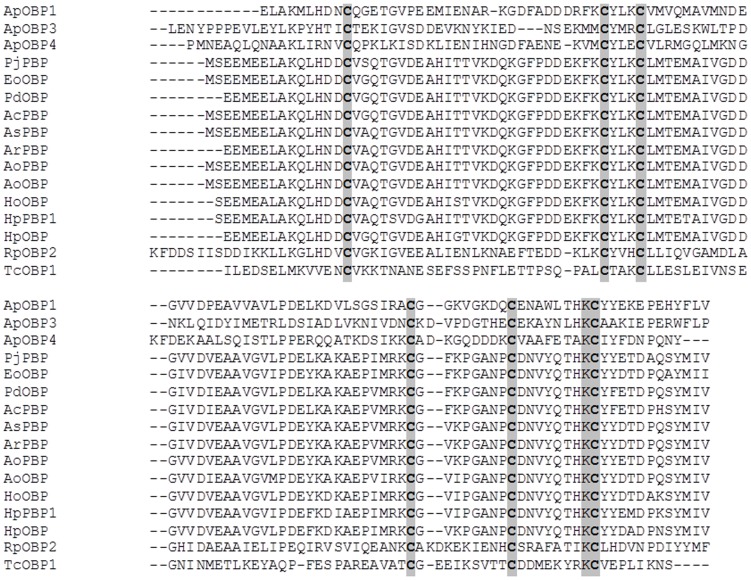
Multiple sequence alignment of three odorant binding proteins (OBPs) of *Agrilus planipennis* with other insect OBPs. Analyses included OBPs and pheromone binding proteins (PBPs) and antennal binding proteins (ABPs) of *Tribolium castaneum* OBP7 (EFA04593), *Anomala cuprea* PBP (BAC06496.1), *Phyllopertha diversa* OBP1 (BAA88061) *Anomala octiescostata* PBP (BAC06497.1), *Popillia japonica* PBP (AAC63436.1), *Exomala orientalis* PBP (BAB70711.1), *Heptophylla picea* OBP1 (BAC07270), *Tribolium castaneum* OBP6 (EFA04594), *Rhynchophorus palmarum* OBP4 (AAQ96921), *Heliothis virescens* ABP (CAA05508), *Agrotis ipsilon* ABP1 (AAP57463.1), *Bombyx mori* ABP (NP_001037500.1), *Anomoala rufocuprea* PBP (BAF79995.1), *Lygus lineolaris* ABP (AAC43033), *Manduca sexta* ABP3 (AF393488_1), and *Agrilus planipennis* OBPs (*Ap*OBP1, *Ap*OBP3, *Ap*OBP4). Identical residues among all the sequences are shaded in grey.

The deduced *Ap*OBP1, *Ap*OBP3 and *Ap*OBP4 proteins showed 6 α-helices (Figure 5A–5C) with a calculated molecular mass of 14 kDa (pI 4.60), 14.1 kDa (pI 4.84) and 14 kDa (pI 7.51), respectively that are within the expected range for insect OBPs [53], [54]. Further, all the OBPs identified in this study revealed a signal peptide in the amino terminus (*Ap*OBP1: first 18 amino acids; *Ap*OBP3: first 22 amino acids and *Ap*OBP4: first 19 amino acids), which supports the fact that OBPs are secreted into the sensillary lymph surrounding the ORNs [53]. The amino acid alignment of *Ap*OBP1, *Ap*OBP3 and *Ap*OBP4 with several other insect OBPs revealed both the conserved and defined spacing of 6 cysteine residues (C1-X_15-39_-C2-X_3_-C3-X_21-44_-C4-X_7-12_-C5-X_8_-C6) (Figure 6) thought to form disulfide bonds that stabilize the three-dimensional structure of the OBP [54].

**Figure 7:**
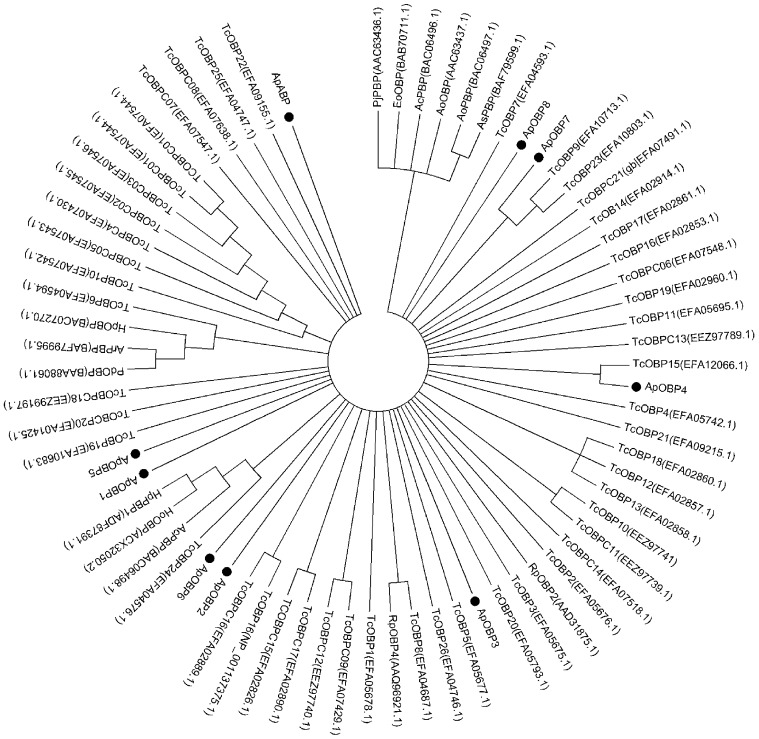
Neighbor joining tree of odorant binding proteins (OBP) from 13 coleopteran species. The signal peptides were removed from each OBPs prior to analysis. All accession numbers are given in parenthesis beside each OBP name. The phylogenetic tree was constructed using MEGA5, using a 70% cut-off bootstrap value.

We also compared all 9 predicted OBPs of *A. planipennis* with 61 coleopteran OBPs in order to reveal their diversity within the insect order (Figure 7). Homology modeling of the *Ap*OBPs revealed close matches with existing insect OBP structures (Table S8), including a conserved number and positions of cysteines involved in disulfide bond formation. Among the 9 OBPs of *A. planipennis, Ap*OBP4 and *Ap*OBP7 are the only ones that were identified as potential orthologs of known OBPs, namely *Tc*OBP15 and *Tc*OBP9, respectively (Figure 7). These observations are in agreement with the diversity of insect OBPs such as in the lucerne plant bug (*Adelphocoris lineolatus*) [55], suggesting the hypothesis of evolution of OBP genes from the same ancestral gene and then divergence by gene duplication after specialization. Since insect behavior is associated with unique odorants (host-specific odorants and pheromones), different species have apparently been under distinct selective pressures, leading to considerable diversification among the OBP members [56]–[58].

**Figure 8:**
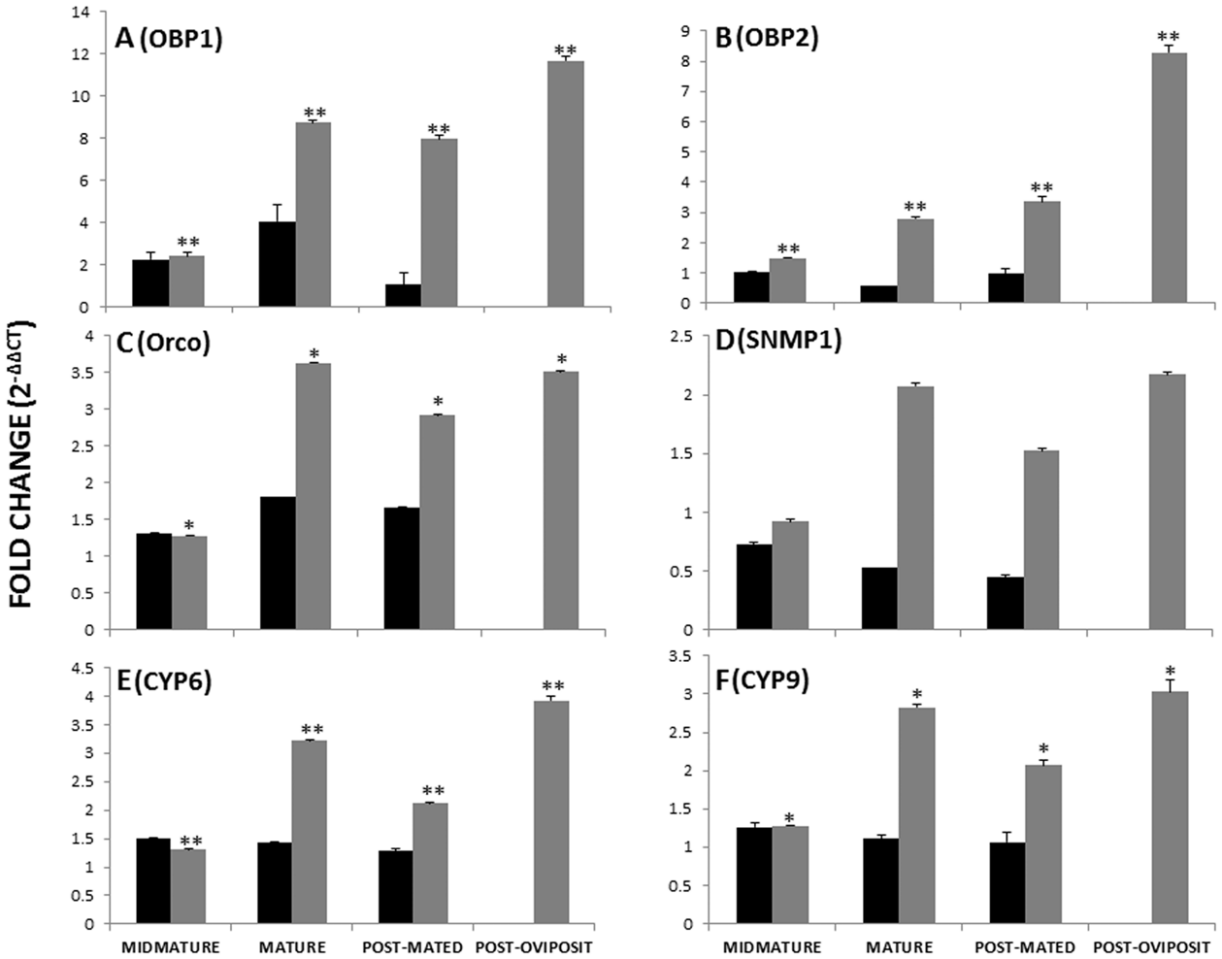
Quantitative RT-PCR analysis of candidate genes involved in *Agrilus planipennis* olfaction. mRNA levels of odor binding proteins, *Ap*OBP1 (A), *Ap*OBP2 (B), odorant receptor, *Apla\Orco* (C), sensory neuron membrane protein, *Ap*SNMP (D), cytochrome P450s - *Ap*CYP6 and (E) *Ap*CYP9 (F) in male (black bars) and female (grey bars) antennae of mid-mature, mature, post-mated and post-oviposit samples of *A. planipennis.* The newly eclosed virgin-male and -female antenna were taken as calibrator to calculate fold change using comparative C_T_ method. Elongation factor-1α of *A. planipennis* was used as an internal control. Standard error of the mean for three technical replicates is represented by the error bars. Single and double asterisks indicate significant differences from the control group at *P* values of <0.01 and <0.05, respectively.

The gene expression levels of odor perception genes (*Ap*OBP1 and *Ap*OBP2) revealed sex- and development-specific patterns (Figure 8, Table S9). *Ap*OBP1and *Ap*OBP2 exhibited significantly higher expression (p<0.01) in females compared to all developmental stages of males (Figure 8A, 8B). Further, *Ap*OBP2 showed an increasing trend in mRNA levels from virgins to post-oviposition females (Figure 8B). Higher transcript levels of *Ap*OBP1and *Ap*OBP2 in females may indicate a perception response to host and/or sex-attractants (aggregation pheromones) as reported in several other insect species [58], [59]. Male-produced sex attractant aggregation pheromones are found most commonly in Coleopterans, and are thought to be important for mate recognition and as short-range pheromones, increasing the chances for mating [59]. Although aggregation pheromones have not been reported in *A. planipennis*, future studies on *Ap*OBP1 and *Ap*OBP2 may help in deciphering their role in host and mate cues (dual perception) as observed with *Bm*GOBP2 of *Bombyx mori* [60].

A similarity search for the 2 retrieved ORs (*Apla\*Orco and *Ap*OR64 ) against the non-redundant nucleotide (nr) database at NCBI using BLASTp, revealed 65% identity with OR83b of *Holotrichia parallela* (AEG88961.1,4e^−134^) and 40% identity with OR64 of *T. castaneum* (EFA10800.1, 1e^−26^ ) at the amino acid level. Phylogenetic analysis of *Apla\Orco* and *Ap*OR64 with other insects revealed a wide divergence (Figure S2) which is in agreement with findings for several insect ORs [61]. We further examined the expression profiles of *Apla\Orco*, an odorant receptor that is highly conserved among insect species [62]. The gene expression profiles for *Apla\Orco* were found to be significantly higher (p<0.05) in antennal samples from female *A. planipennis* compared to males (Figure 8C). The higher expression *Apla\Orco* in females suggests that it is an important receptor for detecting host cues (i.e. locating suitable oviposit sites) or in response to male-produced pheromones, as observed in other insect species [63]. Or83b is known to be required for the function of all heteromeric ORs and the latter are responsible for the initial steps of chemosensation in the ORNs of antennae [64]–[66].

**Figure 9:**
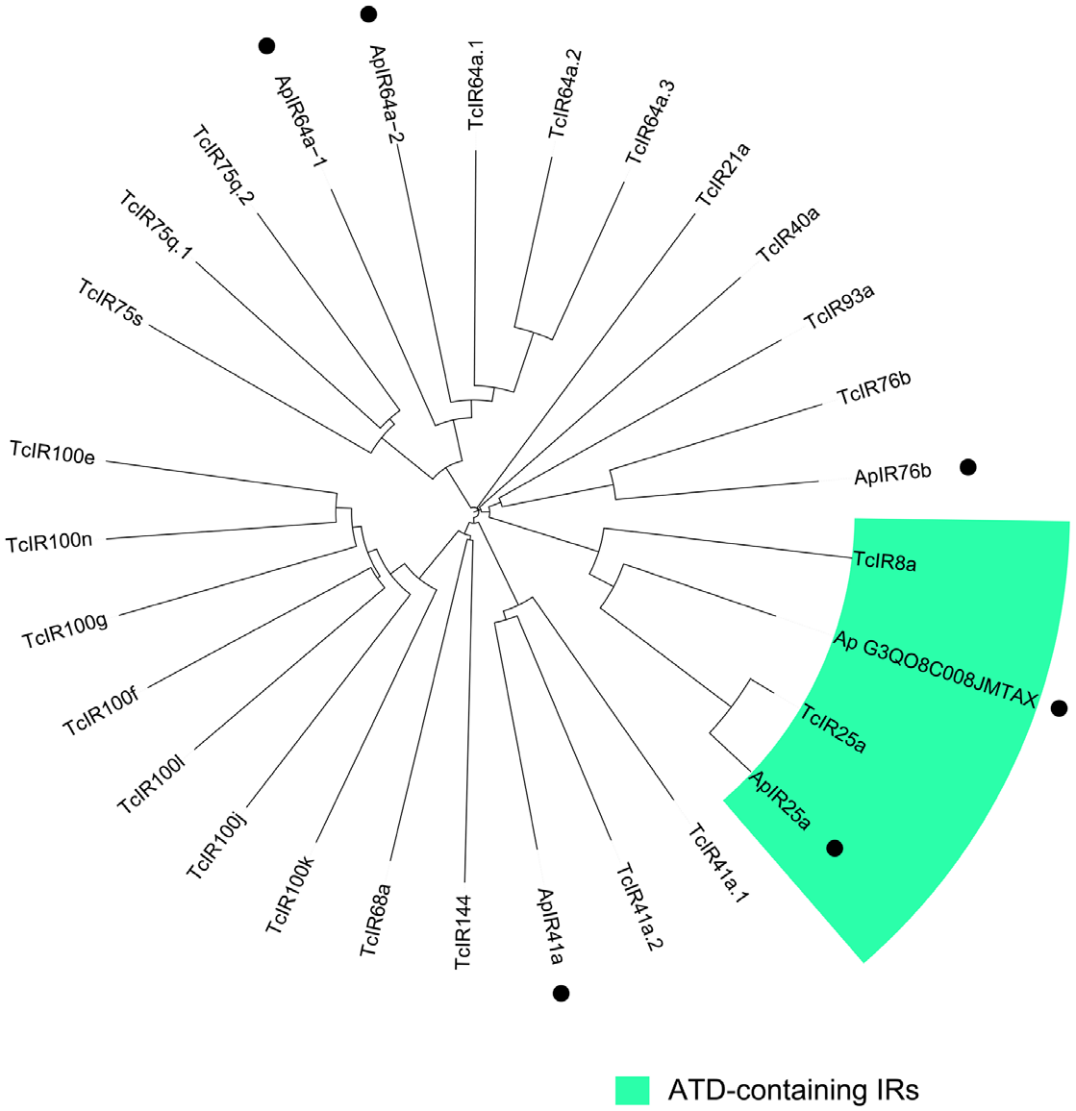
Phylogenetic tree of sequenced *Agrilus planipennis* Ionotropic Receptors (IRs). The tree was constructed using ClustalW including 23 putative *Tribolium castaneum* antennal IRs. Dots indicate *A. planipennis* IRs and the sub-clade of IRs containing an Amino Terminal Domain (ATD) is indicated in green.

IRs are transmembrane proteins closely related to the Ionotropic Glutamate Receptors (IGluRs) involved in neuronal cell-cell communication. All IRs identified to date include in their basic domain architecture a non-contiguous ligand-binding domain (LBD, made of S1 and S2 domains) flanking an ion channel domain [67]. Two single IRs, IR8a and IR25a, also include an amino terminal extension termed the Amino Terminal Domain typical of IGluRs (ATD) [67]. Our sequencing efforts have allowed us to discover 6 antennal IRs, including two with higher sequence similarity to IR8a/IR25a orthologs (*Ap*IR25a and a sequence, *Ap*G3QO8C00JMTAX, which could not be clearly assigned to either the IR8a or IR25a subgroup, Figure 9). The four other IRs of *A. planipennis* include an ortholog of IR41a, IR76b and two orthologs of IR64a (*Ap*IR41a, *Ap*IR76b, *Ap*IR64a-1, and *Ap*IR64a-2). IR41a, IR76b and IR64a are representatives of the repertoire of “antennal IRs” that is conserved among insects, in contrast to a subset of “divergent IRs” that is mostly species-specific [67]. Although the full length sequences of these *Ap*IRs remain to be obtained (none were complete, with the longest contig encoding a 314 aa receptor fragment), IR signature domains were identified in most of the sequences. We also identified 6 *Ap*IGluRs (ligand-gated ion channels) which are thought to mediate chemical communication between neurons at synapses in insects [68]. The conspicuous underrepresentation of gustatory receptors (2 *Ap*GRs, Table 1) is in agreement with several other recent antennal transcriptomic studies [69]–[71]. As their names imply, GRs are expressed mostly in gustatory organs such as the mouthparts (labial palps, glossa, etc.) compared to antennal tissue [72]. In *Drosophila*, a sizable number of GRs are involved in bitter tasting [73]. Future RNA-Seq sensory studies of *A. planipennis* (including the head and/or maxillary palps of both sexes) combined with the genome annotation may better reveal the actual number and sex-biased expression profiles of *Ap*GRs as per other studies [34], [74].

The other non-receptor olfactory gene family members found in the current antennal transcriptome were CSPs (4) and sensory neuron membrane proteins, SNMP (1) (Table 1). Among the 4 CSPs obtained in the current study, we were able to retrieve the full-length sequence for only one CSP (*Ap*CSP4), which had a signal peptide at the amino terminus and revealed the conserved cysteine pattern of C1-X_6-8_-C2-X_18_-C3-X_2_-C4 as observed in other coleopteran insect species (Figure S3) [75]. CSPs are small soluble proteins found in the sensillum lymph of insects, and play a crucial role in binding with odorants and pheromones [76], [77] and are thought to function to a lesser extent in host seeking behavior [77], [78]. The expression profile of *Ap*SNMP was very intriguing, as the transcript levels decreased from newly eclosed virgins to post-mated males. On the other hand, the transcript levels of *Ap*SNMP increased from newly eclosed virgins to oviposition females (Figure 8D). SNMPs are important signaling components of odor detection, wherein combination with other ORs detect specific pheromones from spurious environmental stimuli [79]. These are homologs of CD36, a scavenger receptor important for lipoprotein binding and uptake of cholesterol and lipids in vertebrates, and under-studied in insects [80], [81]. The high expression of *Ap*SNMP in post-oviposition *A. planipennis* females might explain its putative role in host cue detection (Figure 8D). However, future functional studies (RNA interference) will be required to ascertain the actual role of *ApSNMP* in *A. planipennis* olfaction.

The female-biased expression of odor reception genes in *A. planipennis* may be attested to its duration on the host plant. Females tend to spend more time compared to males (Poland, personal observations), which is supported with predominant (76%) female *A. planipennis* on ash trunks [82]. Immature and sexually mature females predominantly feed and rest on leaves. On the other hand, males do spend some time feeding, but then hover in the air around the canopy of trees looking for females to land on. After mating, the males zip off and begin hovering again, whereas the females continue to feed on the host and move down onto the trunk to oviposit (Poland, personal observations). These observations are in agreement with olfactometer assays wherein virgin female *A. planipennis* beetles were most attracted towards beetle damaged or MeJa-exposed ash trees [83]. On the other hand, male *A. planipennis* do not show such responses to insect damaged foliage or MeJa-treated volatiles [84]. Sexual dimorphism is also observed in *A. planipennis*, wherein females tend to be larger and have more rounded abdomens, whereas males are smaller with tapered abdomens [83]. The size dimorphism is likely related to egg production by females. Sensilla on the sternum of males may aid in contact with the female when mounted during copulation. Further, a study on fine structure of antennal sensilla in *A. planipennis* revealed that males seem to have noticeably more uniporous gustatory/taste sensilla than females, suggesting short range/contact cues are important for mate recognition, particularly by males [85]. Therefore, at the current time we are unable to correlate sexual dimorphism with sex-biased expression of odor-reception genes performed in the current study.

The higher number of odor/xenobiotic degrading ESTs (193) compared to odor reception suggests their putative role in odor degradation and detoxification as observed in other insect species [86], [87]. Among the odorant/xenobiotic degrading enzymes, we found a high number of ESTs (83) encoding cytochrome P450s (Table 1), which are thought to participate in detoxification (dietary and insecticides), odor processing (pheromone synthesis and odor degradation), and neuro/developmental functions in insects [88]–[91]. Quantitative expression profiles of putative CYP3 clan members (CYP6 and CYP9) indicated female-biased expression (Figure 8E and 8F) [92]. Both the P450s revealed a constitutive pattern of expression in all the antennal samples of male *A. planipennis*. However, in females, the P450s assayed displayed an increasing pattern from newly eclosed virgins to sexually mature individuals followed by a decline in post-mated insects and then a peak expression in post-oviposition females (p<0.05) (Figure 8E and 8F). This female-biased expression of P450s in *A. planipennis* supports the observation that females frequently encounter host allelochemicals, thus potentially requiring elevated levels of cytochrome P450s.

Besides P450 proteins, the most abundant odorant/xenobiotic degrading ESTs represented esterase's (56), which are thought to function in metabolic resistance and odor degradation in several insect species [93]–[97]. Esterases are also well known for their hydrolytic action on esters during pheromone synthesis and degradation [98]. We also predicted the presence of other important odorant/xenobiotic degrading proteins such as glutathione *S*-transferases (GSTs, 13), aldehyde dehydrogenase (31), epoxide hydrolases (9) and several other antioxidants (Table 1) that have been reported to be involved in odor/xenobiotic degradation [99]. The antennae-specific expression of insect GSTs in moths is thought to play dual roles involving signal termination and protection from toxic compounds [100]. Taken together, these putatively identified odorant/xenobiotic degrading proteins of the current study could play protective roles in the antennal tissue of *A. planipennis.*

## Conclusions

This is the first study of genes involved in the reception, processing and degradation of volatiles in the invasive insect pest *A. planipennis*. The number of odor reception and odor degradation genes identified provides insights into the olfactory processes of *A. planipennis* in detecting host- and mate-cues. The female-biased expression of candidate odorant reception genes will help understand the host- and courtship-cues deployed by female *A. planipennis.* Future studies using homology modeling and molecular docking of the predicted odor reception proteins in combination with RNA interference (RNAi) experiments could reveal critical molecular targets of compounds known to be active toward *A. planipennis* (e.g. host green leaf volatile, (3Z)-hexenol; and the bark volatile 7-epi-sesquithujene).

## Materials and Methods

### Insect rearing

All necessary permits were obtained for the described field studies. Rearing logs were cut at Fox Memorial Park in Potterville, Eaton County, Michigan. We obtained permission to cut the logs from Dan Patton, Director of Eaton County Parks. We also had a permit from the Michigan Department of Natural Resources and Environment (Land Use Permit number WD-FRM-2011-001). It was issued by Don Avers, DNR Wildlife Division.

Naturally infested ash trees were felled at the end of 2010 and early 2011 in Eaton County, MI and cut into 55-cm long logs. Logs were held in cold storage at 4°C until needed, then placed in rearing tubes at room temperature to allow beetles to emerge. Emerging beetles were collected daily, separated by sex, and kept in 295 ml plastic beverage containers with an evergreen ash, *F. uhdei* (Wenzig) Linglesh, leaf in a vial of water for feeding. Up to 5 beetles of the same sex were held in each container and were stored in growth chambers at 25°C with a photoperiod of 14L:10D, and >70% relative humidity. Leaves were replaced three times a week. Newly eclosed virgin beetles (15 of each sex) were sampled between 2 to 3 days after emergence. Mature virgin beetles (15 of each sex) were sampled between 10–12 days after emergence. *A. planipennis* adults require 7–10 days of feeding before becoming sexually mature. An additional 56 sexually mature *A. planipennis* adults of each sex (12–14 days old) were paired for mating on 26 July 2011 and held as individual pairs in separate petri dishes (100 mm diam) with a piece of evergreen ash leaf. Petri dishes were arranged on the laboratory bench in a single layer and each pair was observed every 30 minutes for mating. Pairs that mated and remained in copula for at least two successive observation periods were considered to have mated successfully with sperm transfer. After mating was observed, the pair was placed in a plastic beverage container with an evergreen ash leaf and returned to the growth chamber. Twenty mated beetles of each sex were sampled within 2 days after mating. The remaining pairs of beetles were maintained as individual pairs in plastic beverage containers with fresh evergreen ash foliage and an ash stick (approx. 1 cm diam and 12 cm long) wrapped with curling ribbon as an oviposition substrate for females. Containers containing beetles were held in growth chambers as described above. Leaves were replaced three times a week and ash sticks were checked daily for the presence of *A. planipennis* eggs under the curling ribbon. When eggs were found, females were sampled as post-oviposition females. A total of 10 post-oviposition females were sampled.

### Dissections and RNA isolation

In total 100 antennal pairs (50 male and 50 female adults) from adult *A. planipennis* (newly eclosed virgins, sexually mature virgins, post-mated males and females and post-oviposition females) were cut off and transferred to 2 ml eppendorf tubes for total RNA extraction using TRIzol method. The samples were subjected to QC using the Nanodrop 2000c (Thermo Scientific) before shipping them to Purdue Genomics Core Facility (West Lafayette, IN) for 454 pyrosequencing.

### Assembly, annotation, comparative genomics and sequence analysis

Primary assembly of the 454 sequences of *A. planinpennis* antennae was done using Newbler Assembler (version 2.5). This assembly generated three files: Isotigs (assembled contigs that are possible transcripts), contigs that were not assembled into isotigs and singletons that were not assembled. According to Newbler assembly, contigs with overlapping ends are assembled into contig graphs and branches within these graphs represent possible alternative splicing. Therefore, isotigs, which represent each possible path through graphs, could be considered as transcripts and all the possible paths within one contig graph are called isogroup. Isogroup could be considered as individual gene and isotigs within an isoform are potential alternative splice events of a gene. Contigs and in some cases, singletons could represent exons.

The singletons that were not assembled were reassembled using CAP3 to reduce redundancy with ‘-z’ option set to 1. To avoid redundancy and make as single sequence file, Isotigs, contigs that were not assembled into isotigs, CAP3-contigs and CAP3- singleton were combined using CD-hits [101]. Non-isotigs were defined as all the above sequences except isotigs. For annotation, all the derived sequences were searched against Swiss-Prot (https://www.uniprot.org) and the sequences that did not have matches were searched against National Center for Biotechnology Information (NCBI) non-redundant (nr) database using BLASTx (BLAST 2.2.23+, E-value < e^−5^) [102]. The BLAST results were imported into the Blast2GO suite [103] for annotation of each EST. Blast2GO was used to find conserved protein domains using InterProScan (IPS) [104]. The domain information found using IPS search was merged with GO annotations. The GO terms were exported and categorized using a web-based tool, CateGOrizer with “Aqua” tool [105], [106] To find similarities of the assembled sequences with other species with complete genome sequence, BLASTx searches (*E* value < e^−3^) were carried out against protein sequences of *A. gambiae, A. pisum*, *D. melanogaster* and *T. castaneum.* In addition, the assembled sequences were compared with previously assembled sequences from mid-gut and fat-body transcriptomic sequences with *E* value < e^−6^ and percentage of identical matches above 96% [30]. The secondary structure for all the OBPs were predicted using online tool PSIPRED protein structure prediction server (http://bioinf.cs.ucl.ac.uk/psipred/). Multiple sequence alignment was performed using Clustal W software (http://www.ebi.ac.uk/Tools/clustalw/). Signal peptides were identified using SignalP 4.0 (http://www.cbs.dtu.dk/services/SignalP/) [107] and removed from all OBPs used in the phylogenetic analysis due to their highly diversified nature. A phylogenetic tree was constructed using the neighbor joining method as implemented in MEGA 5.0 [108].

### RACE-PCR

To obtain the full length sequences of *Ap*OBP3 and *Ap*OBP4, 5'- and 3'- (Rapid Amplification of Complementary Ends (RACE) - PCR was performed by the PCR suppression and step-out procedure as per Matz et al. 2003 [109], using nested primers. In brief, cDNA template (prepared from 500 ng RNA of *A. planipennis* adult head), 5prox adapter, 3prox adapter and gene specific primers (Table S9) were used for the first step 5'- and 3'- RACE reactions [1 mM MgSO_4_, 0.3 mM dNTPs, 0.05µM primers, 1µl of diluted cDNA (1:25) with TM buffer (TM buffer: 10 mM Tris-HCl, pH 8.0; 1 mM MgCl_2_) and 1 U of AccuPrime^TM^ *Pfx* DNA polymerase, Invitrogen].The cycling regime of the first stage RACE was: denaturation at 95°C for 3 min, followed by 35 cycles at 95°C for 15 sec, annealing at 55°C for 30 sec and extension at 68° C for 5 min. Final extension was performed at 68° C for 5 min. For the second (nested) step PCR reactions, 5′- and 3′-RACE was performed by paring with universal Udist primers and gene specific forward and reverse primers respectively (Table S10). The second step RACE reaction setup included: 0.1µM of primers, 1.5 mM MgCl_2_, 200µM dNTPs, 1µl of diluted first stage amplified product (1:50 fold diluted 5′- or 3′-RACE product in MilliQ water), and 1 U of Taq DNA polymerase (New England Biolabs). The PCR cycling conditions were: denaturation at 95°C for 3 min, 20 cycles of denaturation at 95°C for 1 min, annealing at 55°C for 1 min and extension at 68°C for 2 min. The final extension step was at 68°C for 10 min. RACE products were resolved on 1.5% agarose gel and purified from the agarose gel as described in Sambrook et al. 1989 [110]. The purified PCR products were cloned into pGEMT easy vector (Promega, Madison) and sequenced at Eurofins MWG Operon facility (Huntsville, AL).

### Quantitative Real-Time Polymerase Chain Reaction

Fifteen pairs of antennae per sample (newly eclosed virgins, sexually mature virgins, post-mated males and females and post-oviposition females) were dissected and transferred immediately into ice-cold Trizol and stored at −80°C until further processed. These samples were homogenized individually in 2.0 ml eppendorf tubes and total RNA was isolated using TRIzol (Invitrogen) following the manufacturer's protocol. Following extraction, all the RNA samples were treated with TURBO DNase^TM^ (Ambion, Inc., Austin, TX) to eliminate potential genomic DNA contamination. The Super Script^TM^ (Superscript III) First-Strand synthesis kit was utilized for cDNA synthesis using 200 ng of total RNA from each sample. All primer sequences used for expression analysis of candidate genes in the current study were designed using Beacon Designer 7 software (Table S9). Standard PCR was performed for all of the primers, and their products were run on agarose gel electrophoresis to ensure single bands. To confirm the tissue-specific expression of all the selected candidate genes (*Ap*OBP1, *Ap*OBP2, *Apla/Orco*, *Ap*SNMP, *Ap*CYP6 and *Ap*CYP9), a routine PCR was performed using pooled antennal cDNA and midgut cDNA as templates (Figure S4). The cycling parameters were 95°C for 5 min followed by 39 cycles of 95°C for 10 s and 60°C for 30 s ending with a melting curve analysis (65°C to 95°C in increments of 0.5°C every 5 s) to check for nonspecific product amplification. A standard curve was done for each set of primers to check for primer efficiency. The curve consisted of 5-fold dilutions over four points. An elongation factor 1-α (*Ap*EF-1 α) was used as internal control gene, which showed stable expression in *A. planipennis* [111]. Fold change in gene expression among male and female antennal samples of *A. planipennis* were derived by the 2^−δδC^_T_ method by taking newly eclosed virgin samples (male and female) as calibrator [112].

### Data deposition

The Roche 454 pyrosequencing reads of *A. planipennis* antenna were submitted to NCBI Sequence Read Archive under the accession number of SRA048250.1 and Isotigs and contigs that are above 200 nt were also deposited in Transcriptome Shotgun Assembly (TSA) database under the submission number of SUB121675.

## Supporting Information

Figure S1 **Comparison of antennal expressed sequence tags (ESTs) with midgut and fat body ESTs of** ***Agrilus planipennis*** **.**
(TIF)

Figure S2 **Phylogenetic relationship of** ***A. planipennis*** **odorant receptors with other coleopteran odorant receptors.** The numbers near branches indicate bootstrap values.
(PSD)

Figure S3 **Schematic drawing of** ***Agrilus planipennis*** **chemosensory protein (** ***Ap*** **CSP4).** Predicted signal peptide is illustrated with an underline. Four highly conserved cysteine residues were shown in grey color.
(TIF)

Figure S4 **Tissue specific expression of antennal genes in pooled antennal (top panel) and midgut (bottom panel) samples.** Genes validated include Elongation Factor 1α (1), odor binding proteins, *Ap*OBP1 (2), *Ap*OBP2 (3), odorant receptor, *Apla\Orco* (4), sensory neuron membrane protein, *Ap*SNMP (5), cytochrome P450s - *Ap*CYP6 and (6) *Ap*CYP9 (7).
(TIF)

Table S1 Length distribution of contigs and singletons obtained in *Agrilus planipennis* antennal transcriptome.
(XLSX)

Table S2 Comparative genomics of assembled transcripts of *Agrilus planipennis* transcriptomic sequences with protein sequences of *Acyrthosiphon pisum, Anopheles gambiae, Drosophila melanogaster* and *Tribolium castaneum.*
(XLSX)

Table S3 Top BLAST hits in the NCBI nr database for each unique contig of *Agrilus planipennis* antennal transcriptome.
(XLSX)

Table S4 **Comparison of** ***A.planipennis*** **antennal transcripts with midgut and fatbody expressed sequence tags of** ***Agrilus planipennis*** **.**
(XLSX)

Table S5 **Gene ontology of** ***Agrilus planipennis*** **antennal transcriptome.**
(XLSX)

Table S6 **Summary of top ten protein domains based on occurrence in antennal transcriptome of** ***Agrilus planipennis.***
(DOC)

Table S7 KEGG Summary of *Agrilus planipennis* antennal transcriptome.
(XLSX)

Table S8 **Transcript and amino acid fasta files of the chemosensory genes profiled in the current study.**
(DOC)

Table S9 **Detailed analysis of** ***A. planipennis*** **odorant binding proteins.**
(DOC)

Table S10 **Primers used in the current study.**
(DOC)
