# Supplementary figures and images for: Identification of Odor-Processing Genes in the Emerald Ash Borer, Agrilus planipennis
Source: PLoS One. 2013 Feb 12;8(2):e56555. doi: 10.1371/journal.pone.0056555 (PMC3570424; doi:10.1371/journal.pone.0056555)

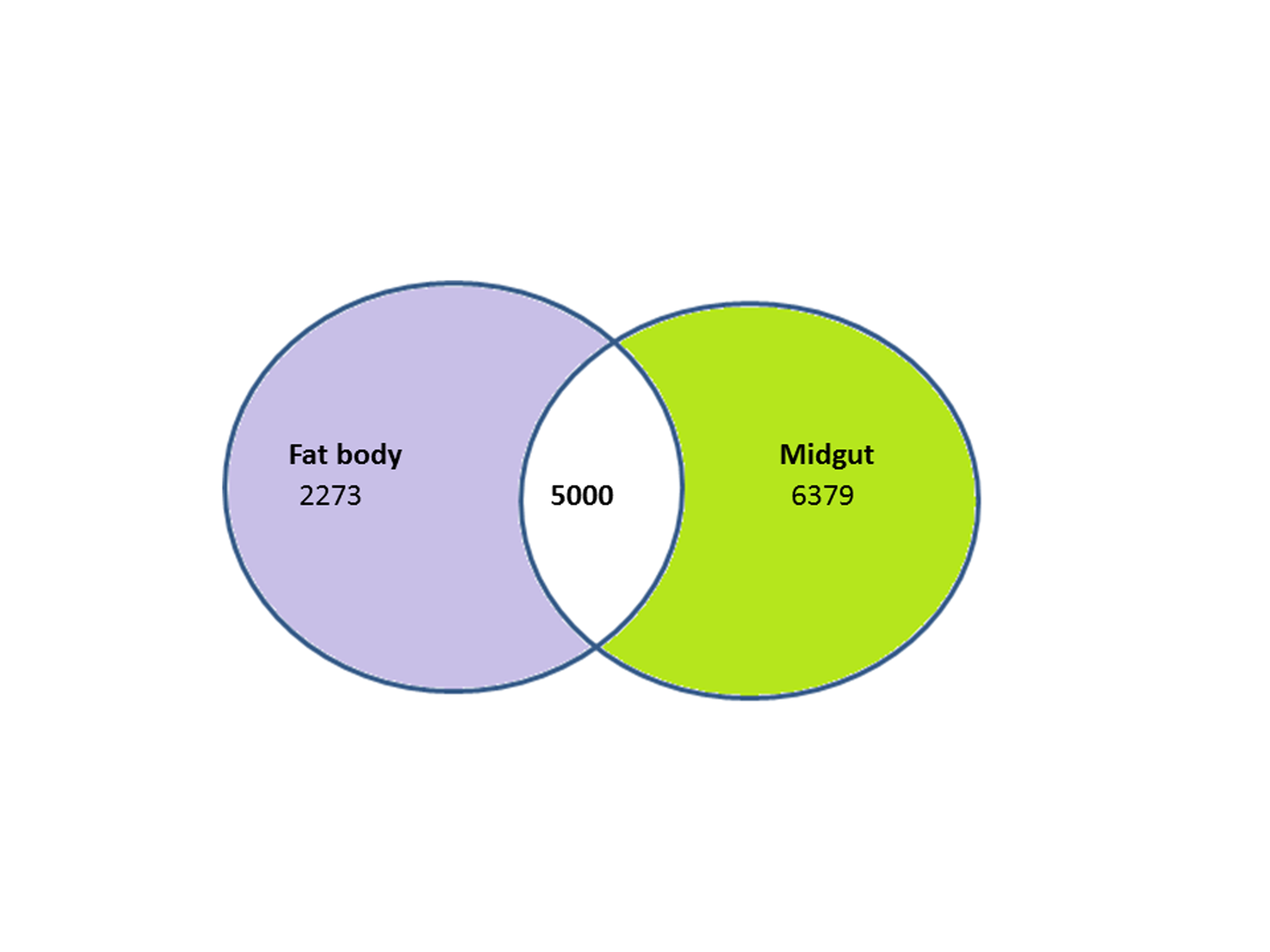

Supplement: Figure S1 — Comparison of antennal expressed sequence tags (ESTs) with midgut and fat body ESTs of Agrilus planipennis . (TIF) [file pone.0056555.s001.tif]

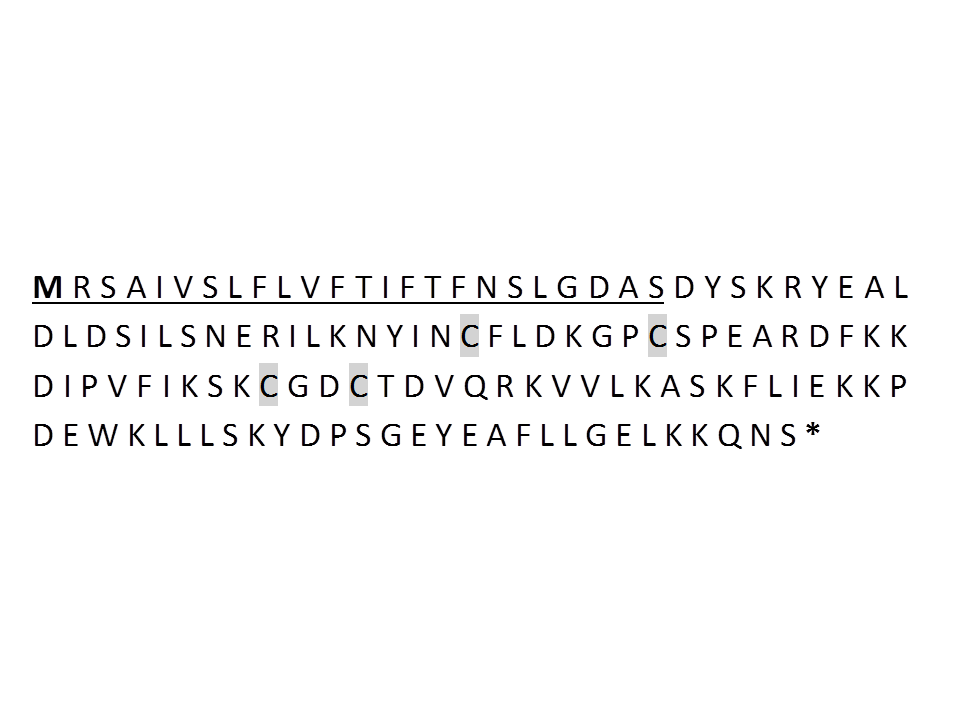

Supplement: Figure S3 — Schematic drawing of Agrilus planipennis chemosensory protein ( Ap CSP4). Predicted signal peptide is illustrated with an underline. Four highly conserved cysteine residues were shown in grey color. (TIF) [file pone.0056555.s003.tif]

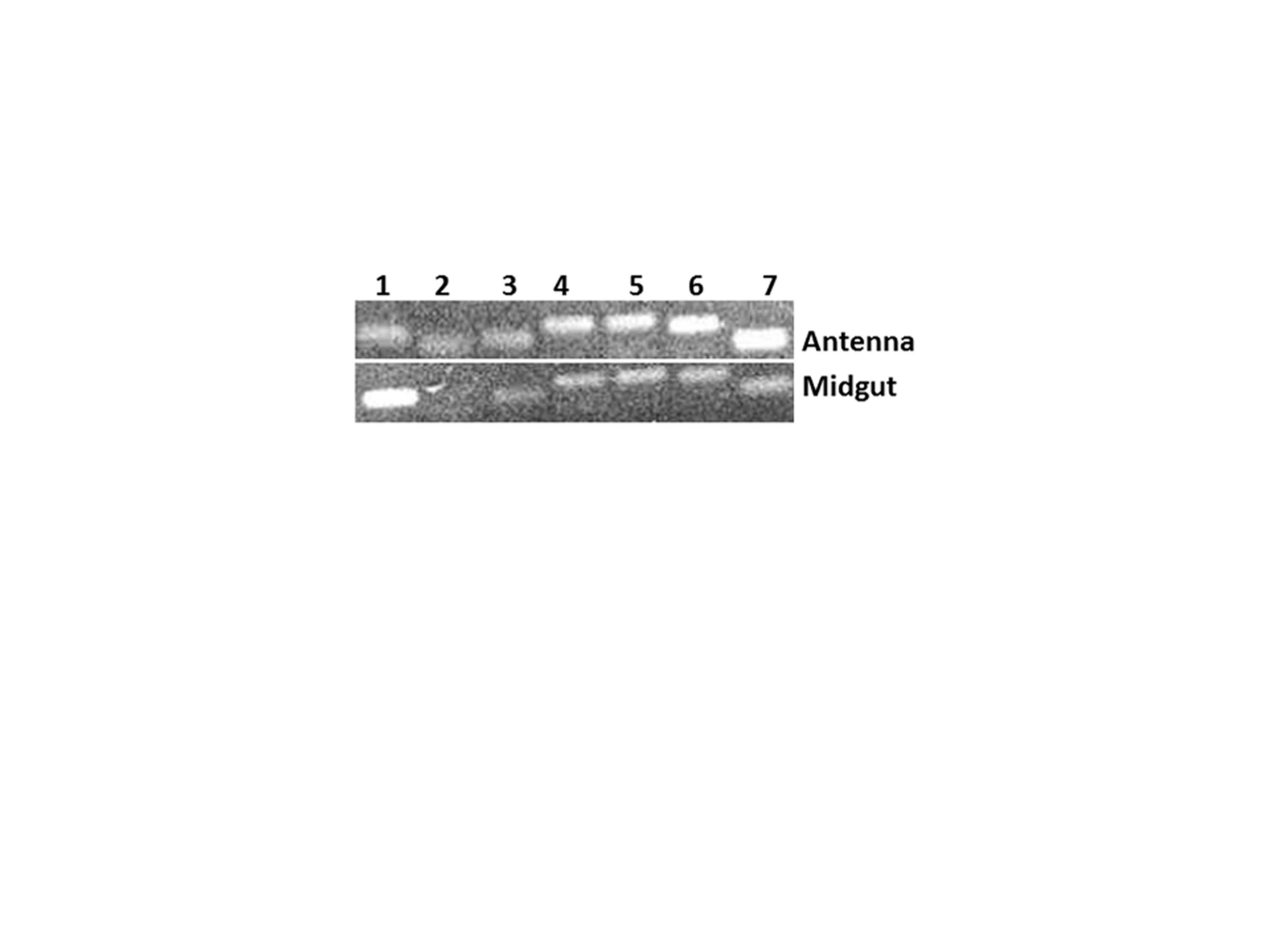

Supplement: Figure S4 — Tissue specific expression of antennal genes in pooled antennal (top panel) and midgut (bottom panel) samples. Genes validated include Elongation Factor 1α (1), odor binding proteins, ApOBP1 (2), ApOBP2 (3), odorant receptor, Apla\Orco (4), sensory neuron membrane protein, ApSNMP (5), cytochrome P450s - ApCYP6 and (6) ApCYP9 (7). (TIF) [file pone.0056555.s004.tif]
