# Supplementary material for: Identification of Odor-Processing Genes in the Emerald Ash Borer, Agrilus planipennis
Source: PLoS One. 2013 Feb 12;8(2):e56555. doi: 10.1371/journal.pone.0056555 (PMC3570424; doi:10.1371/journal.pone.0056555)
Supplement: Table S6 — Summary of top ten protein domains based on occurrence in antennal transcriptome of Agrilus planipennis. (DOC) [file pone.0056555.s010.doc]

***Table S6:*** *Summary of top ten protein domains based on occurrence in antennal transcriptome of* Agrilus planipennis.

| **Domain accession** | **Domain description** | **# occurrence** |
| --- | --- | --- |
| IPR003596 | Immunoglobulins | 314 |
| PR003598 | Zinc finger, C2H2-type | 305 |
| IPR003599 | Protein kinase (Serine/threonine-protein kinase-like) | 298 |
| IPR007110 | WD40/YVTN repeat-like-containing domain | 244 |
| IPR013098 | Armadillo-type fold | 147 |
| IPR013106 | Major facilitator superfamily domain | 123 |
| IPR013151 | NAD(P)-binding domain | 89 |
| IPR013162 | Armadillo-like helical | 68 |
| IPR013783 | Tetratricopeptide-like helical | 54 |
| IPR014756 | Ankyrin repeat-containing domain | 47 |
