# Supplementary material for: Identification of Odor-Processing Genes in the Emerald Ash Borer, Agrilus planipennis
Source: PLoS One. 2013 Feb 12;8(2):e56555. doi: 10.1371/journal.pone.0056555 (PMC3570424; doi:10.1371/journal.pone.0056555)
Supplement: Table S8 — Transcript and amino acid fasta files of the chemosensory genes profiled in the current study. (DOC) [file pone.0056555.s012.doc]

**Table S8: Transcript and amino acid fasta files of the chemosensory genes profiled in the current study.**

| **S.No** | **Gene** | **Nucleotide sequence** | **Amino acid sequence** |
| --- | --- | --- | --- |
| **1** | **ApOBP1** | AGGGTATTTAACAGACATTATATTAATTTATTTTATATTTGTAACTTATTTTAGTAAAAA TAATGAGCGAGTTTTTGTAAAACAATTGTTGCGTCAAATCATCGGAATGGGAATTAAAAA GTAAAAAATATCAATTACAATAATGTTTATTTAATTATTTGCTGAAACAACAGATGTCGA CACCTTCTGCAGGAAAATTGGGTTTTCTTACTAGGGGTACAAATTCTTCAATTTGTTAGA GATTAGACCAGGAAGTAATGTTCAGGTTCCTTTTCGTAGTAACATTTATGAGTTAACCAG GCATTTTCACATTGATCTTTTCCAACTTTCCCGCCACATGCCCTTATACTTCCTGACAAA ACATCTTTCAATTCGTCGGGTAGAACAGCAACCACCGCTTCAGGGTCCACTACTCCTTCG TCATTCATCACTGCCATTTGGACCATGACGCACTTTAAGTAGCATTTAAATCTATCGTCA TCGGCAAAATCACCTTTGCGAGCATTTTCTATCATTTCTTCAGGTACTCCTGTTTCCCCT TGACAATTATCATGGAGCATTTTTGCTAATTCCATCATCTCCTCGGATATGCACAGGATA AACGGTAAAACGGCAATCAATGCAAACTTGAGCCACATTTTGCGAATGTTTCTGTTATTA ATCTTCTCTGTGCACTATTTACGTATGAATGAATCAATAAAAACAAAATTTTTGATTTTA TTGCAGTAATATTATAGACAATTCT | ELAKMLHDNCQGETGVPEEMIENARKGDFADDDRFKCYLKCVMVQMAVMNDEGVVDPEAVVAVLPDELKDVLSGSIRACGGKVGKDQCENAWLTHKCYYEKEPEHYFLV |
| **2** | **ApOBP2** | TTAATTAATTGCTATTGCATTTTATTTTTAAGTGTACTGTATTTGAAATTTACATACTAT  AACATTTTTATTAAGTAAATAGGGTATAATTTTAAATTCGGGATTGATTTAATTTTCTGG  AGGTGGTGGATCAAAGTGGTGTGGAAATTTTCCATTCTTAATCTCATCAATCATAGTCTC  ACATTTAGCTTTGTCTTTCACTTGATCTGCTGGACAATTCTCAAGAGATCTGAAGAAGAG  ACAATTAGATAGAAACAGGGGCTTTGGATTACACTTGGATTCTTTCGGAATTTCTTTTTC  GATATCTTCAATGCACTTCTTAGCTGCTTCTTCGATGTGATTTTCCATAAATGTTCCATT  TATTTTGCCTTGCGCGACTTCAAGTAATTTGGCTAACACAATATTTCCATCACTGTCTAA  CAATTCGTATTTTTTAGCAGTACACTCTTCAATACAAGCAATTTTTTCTGCCAGTTCTTC  CAATTCTTGATCGGTTGGCGGATTGCTGTGATTCATACTTTTTGGATCGCCCAAAATTTC  ACTTATACATTCTTTCATTTTTTCGTCTTCTGGGGCAGCCATAAACGGTTCAAATTCGCA  GCATACCTCAAAAATAGGTGCATGGTGCCTGTTCTCAGAAAAAGAGTTTGCAACAGCAAA  CCCGACAATAAGCAGCGGAACTACCAACGAAGAACTCATGTTGTTGAAAACTAGTTACGC  ACTATT | [M](http://web.expasy.org/cgi-bin/translate/dna_sequences?/work/expasy/tmp/http/seqdna.12303,4,10) S S S L V V P L L I V G F A V A N S F S E N R H H A P I F E V C C E F E P F [M](http://web.expasy.org/cgi-bin/translate/dna_sequences?/work/expasy/tmp/http/seqdna.12303,4,49) A A P E D E K [M](http://web.expasy.org/cgi-bin/translate/dna_sequences?/work/expasy/tmp/http/seqdna.12303,4,57) K E C I S E I L G D P K S [M](http://web.expasy.org/cgi-bin/translate/dna_sequences?/work/expasy/tmp/http/seqdna.12303,4,71) N H S N P P T D Q E L E E L A E K I A C I E E C T A K K Y E L L D S D G N I V L A K L L E V A Q G K I N G T F [M](http://web.expasy.org/cgi-bin/translate/dna_sequences?/work/expasy/tmp/http/seqdna.12303,4,127) E N H I E E A A K K C I E D I E K E I P K E S K C N P K P L F L S N C L F F R S L E N C P A D Q V K D K A K C E T [M](http://web.expasy.org/cgi-bin/translate/dna_sequences?/work/expasy/tmp/http/seqdna.12303,4,185) I D E I K N G K F P H H F D P P P P E N |
| **3** | **ApOBP3** | AAAAGCAAAATTCTATTTCGAAAAGTACAACACACATTTGCGTTACTTATAACTACAAAA CTAATTCTAATTTATACTGTTTATGTACGTAATACAATATAATAATCATAAGTAGGGTCC GTAGCATTCATTGTCATGTATTTTTCCATCCAAAAAATATGAATAGGGTGTGCAATATCA AGGCAAAAACCATCGCTCAGGTTCAATTTTTGCTGCACATTTATGTAAATTGTAGGCTTT TTCACATTCATGGGTACCATCAGGAACGTCTTTACAATTATCCACAATATTTTTTACCAA ATCCGCTATAGAATCAAGACGTGTTTCCATAATGTAATCGATTTGAAGTTTGTTATCAGG AGTAAGCCATTTGGATTCCAAACCTAAACATCTCATATAACACATCATTTTTTCTGAATT ATCTTCTATTTTATAGTTTTTAACTTCATCATCCGAGACGCCAATCTTTTCGGTACATAT AGTATGGTACGGCTTCAGATATTCCAAAACTTCAGGGGGAGGATAGTTTTCAAGCAAAGC GTTTGTATTTGTATAATAACAAAACAACAGCACAGCAGCTACAAAATATGTGACTGTAGT CATTGTAGGATGGATCTTACAGTTGAATAGAGGGATAAAATAATTTTTCC | LENYPPPEVLEYLKPYHTICTEKIGVSDDEVKNYKIEDNSEKMMCYMRCLGLESKWLTPDNKLQIDYIMETRLDSIADLVKNIVDNCKDVPDGTHECEKAYNLHKCAAKIEPERWFLP |
| **4** | **ApOBP4** | ATGAGAATTTTTCTTTTGTTATTAAGTTGTGCTTTTAGTTTTAATAGAATTGAAGCTACG  CCAATGAACGAAGCTCAACTTCAGAATGCTGCAAAACTAATTAGAAATGTTTGTCAACCT  AAACTTAAAATATCCGACAAACTGATAGAAAATATACACAATGGCGATTTTGCTGAGAAT  GAAAAAGTGATGTGTTATCTCGAGTGCGTTTTACGTATGGGACAGTTGATGAAAAATGGG  AAATTTGATGAAAAGGCAGCATTATCCCAAATTTCCACTTTACCTCCTGAGAGACAGCAG  GCCACAAAAGATTCGATAAAAAAATGCGCCGATAAAGGGCAAGACGATGATAAGTGCGTG  GCAGCGTTTGAAACTGCAAAATGTATTTACTTCGATAATCCACAGAATTACTTTTTACCG  TAA | PMNEAQLQNAAKLIRNVCQPKLKISDKLIENIHNGDFAENEKVMCYLECVLRMGQLMKNGKFDEKAALSQISTLPPERQQATKDSIKKCADKGQDDDKCVAAFETAKCIYFDNPQNY |
| **5** | **ApOBP5** | TCCATCGTTGTTCTTTATTTAATTTCTACTAATGCAGATCCTGGGAAATCGAGTACTATC GAAATTCGGGAATGGGAAAATAATTTGATAAAGGACGAACTGTCATGCATAAATAGCACAGGGGTAAGTTTAAGCGTGATAGAAAGAACAAAAGTTACTCTAGAATTACCAGAAGACGACCCAAAGTATAAAGAATATTTAAAATGTTTTTATACGAAAAGAGGCTATCAAAGCGATTCAGGGGAAGTTCTTTATGACAACATCAAGATAATGATACATCAATTTACTAACGCAACTGAAGCGACAAGAATAATAGACTTGTGCAAAGAAATGAGAGGAGCAACTGCT | SIVVLYLISTNADPGKSSTIEIREWENNLIKDELSCINSTGVSLSVIERTKVTLELPEDDPKYKEYLKCFYTKRGYQSDSGEVLYDNIKIMIHQFTNATEATRIIDLCKEMRGATA |
| **6** | **ApOBP6** | TGGTACTTTTTCTTTTTAGAAGATAAAGTATTTATTTAACCACTAAATACAATTTACGAT TTTTTGTACTAATTTTAATATATATTACATTACATTACATTAAAAAACCAAGCAAATTCA AGGGGTGAACATTCTTTTTATTGCTTTACGTACTGATGACAACGTTTAGCTTTACAATGT TCATGTTTTCGTCCTTTAAGTTTACTTAATTTTCTTCAGTAATATATATTAGGAAAGTTT GTCATACATAAATACATAATTATTTCGAAACACTAATTAATCAATAATTACTGAATAAAA TAATATACATAGCATTATTCCAGATGCTACCATTCTTTACGGTAAAAAGTAATTCTGTGG ATTATCGAAGTAAATACATTTTGCAGTTTCAAACGCTGCCACGCACTTATCATCGTCTTG CCCTTTATCGGCGCATTTTTTTATCGAATCTTTTGTGGCCTGCTGTCTCTCAGGAGGTAA AGTGGAAATTTGGGATAATGCTGCCTTTTCATCAAATTTCCCATTTTTCATCAACTGTCC CATACGTAAAACGCACTCGAGATAACACATCACTTTTTCATTCTCAGCAAAATCGCCATT GTGTATATTTTCTATCAGTTTGTCGGATATTTTAAGTTTAGGTTGACAAACATTTCTAAT TAGTTTTGCAGCATTCTGAAGTTGAGCTTCGTTCATTGGCTGAAAATATCGTCGTATAAT TACAAATTAGAATTCACCCTCACCAAGATTTATAATCAGCAAATTACTCTTGACTAATGT TTTGTCATTATTTAGTCACCATGAGATATATTTATCTAAAATTTTCAATCTAAATAGTTT ATAATATAGATGCATTAAATATAATTTAAGAGTCATTAATACAAAATAAAAA | TPMNEAQLQNAAKLIRNVCQPKLKISDKLIENIHNGDFAENEKVMCYLECVLRMGQLMKNGKFDEKAALSQISTLPPERQQATKDSIKKCADKGQDDDKCVAAFETAKCIYFDNPQNYFLP |
| **7** | **ApOBP7** | AGAAGAAGACATTTAGCTTTTAATAGATATTTACTCTTATAACAGTATCTAATGTTTGTT GCACTATGATTATTGCCAATAACAGTACATTTTATGAAATTTCAGTACATAAATCATAAC GATTCAGTTCATGCATTTATTAAAAGAGAAATATCTTAAATTAAACTGAATTTAGTTTCG TTGTAGACGCATTTGTAAATTGCTTCTGCCGATTCCTTTTTGTCTAAATTCTTCTGGGAG GTACATTTTGCAACAAGTTTCTTCGCATTTTCGGCAGCAGTTACTGATGCTAGTTTGGTT TCAAGTACGGCTGTCTGGATTTTTCCATCGGGTGTAATAAATCCAGATTTTTCAATGACG CAATACAAGTAATCCTTTAGAGTTTTATTATCTGCAAGATCCCCTTTGCGTGCTTTTTCA AGAAGTAGTTTATCTGCTGGAAACGATTTTAGACATTCGTCCTGGTGTTGCTTTAGCTTA GCTTTTTGCTCGTCAGTCATGTCGCCTGAACTGTGGCAATTACAG | MTDEQKAKLKQHQDECLKSFPADKLLLEKARKGDLADNKTLKDYLYCVIEKSGFITPDGKIQTAVLETKLASVTAAENAKKLVAKCTSQKNLDKKESAEAIYKCVYNETKFSLI |
| **8** | **ApOBP8** | TTAATTAATTGCTATTGCATTTTATTTTTAAGTGTACTGTATTTGAAATTTACATACTAT AACATTTTTATTAAGTAAATAGGGTATAATTTTAAATTCGGGATTGATTTAATTTTCTGG AGGTGGTGGATCAAAGTGGTGTGGAAATTTTCCATTCTTAATCTCATCAATCATAGTCTC ACATTTAGCTTTGTCTTTCACTTGATCTGCTGGACAATTCTCAAGAGATCTGAAGAAGAG ACAATTAGATAGAAACAGGGGCTTTGGATTACACTTGGATTCTTTCGGAATTTCTTTTTC GATATCTTCAATGCACTTCTTAGCTGCTTCTTCGATGTGATTTTCCATAAATGTTCCATT TATTTTGCCTTGCGCGACTTCAAGTAATTTGGCTAACACAATATTTCCATCACTGTCTAA CAATTCGTATTTTTTAGCAGTACACTCTTCAATACAAGCAATTTTTTCTGCCAGTTCTTC CAATTCTTGATCGGTTGGCGGATTGCTGTGATTCATACTTTTTGGATCGCCCAAAATTTC ACTTATACATTCTTTCATTTTTTCGTCTTCTGGGGCAGCCATAAACGGTTCAAATTCGCA GCATACCTCAAAAATAGGTGCATGGTGCCTGTTCTCAGAAAAAGAGTTTGCAACAGCAAA CCCGACAATAAGCAGCGGAACTACCAACGAAGAACTCATGTTGTTGAAAACTAGTTACGC ACTATT | NSFSENRHHAPIFEVCCEFEPFMAAPEDEKMKECISEILGDPKSMNHSNPPTDQELEELAEKIACIEECTAKKYELLDSDGNIVLAKLLEVAQGKINGTFMENHIEEAAKKCIEDIEKEIPKESKCNPKPLFLSNCLFFRSLENCPADQVKDKAKCETMIDEIKNGKFPHHFDPPPPEN |
| **9** | **ApOBP9** | ATTACAACATGAGCGTTTATTATTTATAGGTAGATAAAGTGATAGTTTTTATTATAATTG ATTTAACAGAACGTGATAATCCAGCCTGGTCAAGTGTCGGCATTCATTTGTGCCCATTGC CGTGGAAAAACGGCAAATAGACATTGGTAAAAAGTTTTTAACCTTTTCACATATATCGGA TCCTCTGTTCTCCTTACAGTTTTCAGGCATAGCGTTCCACATTTTTTCGGGAAAGTTCTT TTGCATTACATTTCCATCTTCGTCAACAAATCCACGGCCCTTTATCACGCATAAGACGTT CTCGCAAACATTCGGGTCATCGGCTTCAGACGGATATATTCCTTGCTTTAAACTTTCAAC GGTCTCTTTTGAAACAGGGAATTTCTCTTCGCAAATTTTTCTCATTTCCAGTAAATGTTG AATATCCTCTTCGAACGGATTTACCTCTTCAGCAGCAACATTACCCAAGAACAATGCTAC CAATACAATAAAACCTAAACGAAACATTGTTGTGTTCACGCTACTTCTTGTTATAAATG | EEVNPFEEDIQHLLEMRKICEEKFPVSKETVESLKQGIYPSEADDPNVCENVLCVIKGRGFVDEDGNVMQKNFPEKMWNAMPENCKENRGSDICEKVKNFLPMSICRFSTAMGTNECRHLTRLDYHVLLNQL |
| **11** | isotig01857*-Ap*IR | GACTTCTTTAACACCCCAAGGAGGAGGTGAAGCGCCAAAGAATCTATCGGGACGATTTGT  AGCAGCCACTTGGTGGTTATTTGGCTTTATTATAATTGCTTCGTATACTGCCAATTTAGC  AGCGTTTTTAACCGTTTCTCGATTGGATACTCCCATCGAATCTCTTGACGACTTATCGAA  ACAATATAAAATCCAATACGCACCCACGAAAGATTCATCCACGATGACTTATTTTGAACG  AATGGCTAACATTGAAGATCGATTTTTTGAAATATGGAAAGACATGAGCTTGAATGACAG  TTTAAGCGATGTCGAACGTGCTAAACTGGCTGTATGGGATTATCCTGTGAGCGATAAGTA  CACAAAAATGTGGCAGGCTATCAAAGAAGCTGGAATGCCAGCAACGTTGGAGGAAGCTGTTAAAAGAGTACTACAATCGAAATCGTCCAGCGAAGGATTTGCTTACCTAGGTGATGCGACTGACATAAGGTTTCTCGAACTTACCAACTGTAATCTGCAAATGGTTGGAGAGGAATTTTC  TAGAAAACCTTATGCCATTGCTGTTCAACAAGGCTCTCCTTTAAAAGACCAATTCAATAC  AGCAATTCTGACGCTTCTCAACAAAAGACAATTAGAAAGATT | TSLTPQGGGEAPKNLSGRFVAATWWLFGFIIIASYTANLAAFLTVSRLDTPIESLDDLSKQYKIQYAPTKDSSTMTYFERMANIEDRFFEIWKDMSLNDSLSDVERAKLAVWDYPVSDKYTKMWQAIKEAGMPATLEEAVKRVLQSKSSSEGFAYLGDATDIRFLELTNCNLQMVGEEFSRKPYAIAVQQGSPLKDQFNTAILTLLNKRQLER |
| **9** | isotig00923*-Ap*IR | CTGTTACCATTGGAAGTATTCAATGGCATATCCTTATGAAAGGACCGAAGCTTTCGGCTA  ACGGTTCTGGTCTTTTACAACCATTTACTTGGGAGGTTTGGATTTTAGTTGTAATAACGC  TTTTAGTGGTTGGAGCTGCAATGTATTTTATTGCTTTTATTCAGTCAAGATTTGAAAAAA  GAGTTGACAAAAAAATGTTTTCTTTGCAATCTTGTATATGGTTTGTCTATGGAGCCATAT  TGAAACAAGGAAGTACTTTGAATCCTGTAACAGACTCTTTGAGAATAATATTTGCAACAT  GGTGGTTTTTCATAATGATTCTTACTGCATTTTACACTGCAAATTTAACGGCATTTTTGA  CTTTAAACACTTTTACATTACCGATCAATAATATTAGAGATATTGGAAGGAAAGGATATA  CATGGTGTACACAAGTGAATGCGGTGGATAATATAACAAACGATCCAACTGAAGAGTTGT  CGAAGGCATTAGTTGGTTCAACTAAAAAAGTGGTAAATACTTCTGATACAATAAATTTCA  TGAATAACTACGTTATTGGAAAAAATTATTTGTTTATTGCCGAAAAGCCCCTGATAGATA  TTTGGTTGTATAAAGCGTATGTGAAGAAAGTTGCATTTAATCCAAATGATGTTCTGTCAG  ACTGTCCATTTGCTGTTACCGATTGGAGTTTACTTACAACATCAATTGCTTTTGCTTACT  CTAAAAAATTTACGTACACCTTCTTGTTTGACAAAGCTTTACAACGACTGGTCGAAGCCG  GCTTAATTAACTTCTGGTTCAGGAATATGTTACCGAGAGCTCAAATATGCCCTTTAAATC  CCAAAGTAAACGAGAGACGATTAAGAAACAGCGATCTGCAACTGACTTATTACATCGTAG  CAGTCGGTTTTGCTATAGCAGCTCTTTTTTCTGTGCTGAATTGGTAAATAATATAATTAA  AAAGAGACGGCATACAAGTATTGTGACGATCCCGCAAAATATGTTATTTACGAAATCTCC  ACCGCCGCCATATAGCTCTCTATTTGGACCTCCATTTCCTAAGGAACATAATTACAAGGT  GAAACGGAAAGAAATTAATGGACGAGATTACTGGTGTGTAACTTCGGTTAATTCAAAAAC  AACGAAACTTATTCCTGTAAGAGCGCCTTCGGCTGTTCTGTTTAATTACACAAAATAC | VTIGSIQWHILMKGPKLSANGSGLLQPFTWEVWILVVITLLVVGAAMYFIAFIQSRFEKRVDKKMFSLQSCIWFVYGAILKQGSTLNPVTDSLRIIFATWWFFIMILTAFYTANLTAFLTLNTFTLPINNIRDIGRKGYTWCTQVNAVDNITNDPTEELSKALVGSTKKVVNTSDTINFMNNYVIGKNYLFIAEKPLIDIWLYKAYVKKVAFNPNDVLSDCPFAVTDWSLLTTSIAFAYSKKFTYTFLFDKALQRLVEAGLINFWFRNMLPRAQICPLNPKVNERRLRNSDLQLTYYIVAVGFAIAALFSVLNW |
| **10** | isotig02106*-Ap*IR | AAGAAATAATACAGAACCTCCAAAGTCAACTTCTTTTCTTTGAAGGGCTCCTGTCATACC  ATCAAAGGAACCGTTTGCAAGTTTAAAACCCCAAGAATCGGTTAAACTAAAGTTTAATGA  GAAATTGTAAATTTCTTTGCAGTGATTCATAAGTACACTGTGAAACTTGGTACGAGAATC  GACCCTATAATTATTTTCTTTCAAAATGTATTCTTCTAATTCACCCTCGAATTTATTAAC  CAACACTGCAATTGTTTTAAATACAACGCCACTCATATTTTTTCGGTTCCAATATTTATT  GTAATTGTTTGATTTTAAATTAAATGAAAAATTATTCGATTTTTTACTAGCACGTTGTAA  TTTAATTTCCCCCCTTTTTGTAAGCGGCATTGTACACATCTATCAACGTCACATTTACAT  TAACTCCGTTTCCGTATTCGCATTCCGCCACTAACAGATTTGAATTTATATTTGTATTTA  CATTAGAAAAAACTTTTTTAATAAATTTCTTTGTGATTATCAACCAGTGATAGGAATCGT  TAAAAAGTTCAAATTCGCCAGC | MCTMPLTKRGEIKLQRASKKSNNFSFNLKSNNYNKYWNRKNMSGVVFKTIAVLVNKFEGELEEYILKENNYRVDSRTKFHSVLMNHCKEIYNFSLNFSLTDSWGFKLANGSFDGMTGALQRKEVDFGGSVLFL |
| **11** | G3QO8C008JMA0O_ *Ap*Ir | TCAAAAAATTGGCCACTGGCATAACAAAACTTCTAACAAAATAAAGTATAAGTACGGAAAATTTCATAGAAGACGAAATATGTCGGAAGTAAAATTTAAAACAGCGCTAGTGATCACATTAAATGGTACCGTTGATGTTGATAAATATGCACTTGATGAAACCAGTCGTCAAAACAATACTCACAGTAGATACCACAGTGTCGCAATGAGAATCTGCAGGGATTACTATAATTTTTCAATCGAATTGATGGGCACAAACTCATGGGGTTACGAAAAGTCTAATGGCACATTTGATGGATTAGTAG | QKIGHWHNKTSNKIKYKYGKFHRRRNMSEVKFKTALVITLNGTVDVDKYALDETSRQNNT  HSRYHSVAMRICRDYYNFSIELMGTNSWGYEKSNGTFDGLV |
| **12** | G3QO8C008JMTAX_ *Ap*Ir | GGGAAAACGTTATCGACTTTACAGTTCCATTTTACGATCTCGTCGGAATTACAATTCTTA  TGAAACTTCCGGAAACTCCAACATCGCTATTCAAATTTTTAACAGTTTTAGAAAATGACG  TGTGGCTTTGTATTCTTGCGGCATACTTCTTTACAAGTTTTC | ENVIDFTVPFYDLVGITILMKLPETPTSLFKFLTVLENDVWLCILAAYFFTSF |
| **13** | G3QO8C008JLD8B*-Ap*IR | CTTACATTATCCTCCATTTCATATTGTGGAGGACCCCGCTAACGAATCTGTTGGAGCGGA  AACAAATTTCGCTAAAGAATACGCTAAAAGGCATAATATGAGCGTGGAATATGTCATCGA  CGAAGTGGGACAATGGGGAAACATTTATGACAACTGGACAGGGGATGGTTTAGTTGGAAATACTGCAATGGATAACGCCGATATCGGCTTCGGTGCGGTCTACCTGGAATGGTTTCCATT  GTATTCGTTTTTGGATTTTTCACATCCTTGCATTCGTTCAGGAGTTACTCTCTTGGTTCC  AGAACCAATTTTAGCCGGAGGTTGGACAGTTCCTTTATTTTCATTTTCAACTGAAATGTG  GATTGCGACATCTATATCCTTCGTATGTACCA | LHYPPFHIVEDPANESVGAETNFAKEYAKRHNMSVEYVIDEVGQWGNIYDNWTGDGLVGNTAMDNADIGFGAVYLEWFPLYSFLDFSHPCIRSGVTLLVPEPILAGGWTVPLFSFSTEMWIATSISFVCT |
| **14** | isotig00749  ***Ap*OR83b** | AAGTAACAGTGACAAAAACAAGAGAAAAAATAGAAATTTAAAAAAGTACGGTTTAGTGGCGGACCTAATGCCTAACATTCGTCTAATGCAGGCTTCTGGTCATTTTTTATTTAATTATTA  CGACAACATGGGGGGTATGATGCACTTAATAAGGGTTGGCTACTGCTG GACGTTGTTGAT  TCTACTTCTTTTCCAATTTGGGGGCGCATTTGGAAATTTAATTGTAGAAGCTGACGATGT  AAACGATTTAGCAGCAAACACAATAACAGTACTATTTTTCACCCATTGTATTACTAAATA  TGTTTACTTTGCAGTACGATCTAAACTATTCTACAGGACATTCGGTATATGGAATCAAAG  TAACAGTCACCCCCTGTTTGTTGAATCTAATAATAGATACCATGCTGTGGCACTAAAGAA  AATGAGAATACTTTTATCGGCTGTCGTAGCTTTCACCGTTTGTAGTGCAATTGCGTGGAC  AACACTGACTTTTTTTGACGAAAGCGCGAGGGAAAGACCCGACCCCGAAAATGAAAATATCACCGTTACGACACAACTCCCTAAATTGCTAGTAAAGTCCTGGTTACCCTATAATGCTAT  GTCAGGGATGAAGCATTCCCTAACATTTGGATTTCAGGTGTATTACGTATTTATTTCCAT  GATACATGCCAATTTGATGGATGTCCTTTTGTGTTCCTGGCTGATTTTTGCATGCGAGCA  ATTAATGCATTTGAAGGAAATTTTAAAACCCCTCATGGAATTGTCTGCAACGTTAGACAC  ATACGTCCCCAAAACGGCTGACCTATTTAGAGCCCCCAGTACCAAATCGCAAGACAAACTTATCGAAAATGATTACAACGAAAAAATAGCGATGATTTAGGAGGAGATTCTTATTACATAAATAACCAGGAATTCAATGTCCACTATCGCAGTGGGGCATTGCAAACGTTTGCATCTGGTGGTGGGGGTATCGGACCCAATGGTCTTACTAAGAGGCAGGAGCAAATGGTCAGATCTGCTATTAAATATTGGGTCGAAAGGCATAAGCATGTCGTAAGACTAGTCACCGCAATCGGGGACAGTTATGGGGTAGCTTTACTATTGCATATGTTAACATCTACAGTAATGCTAACTCTTCTTGCGTATCAGGCCACAAAGATTACTGGCGTTACCCCGTATGCAGCGTCTGTACTGGGTTACCTGGTCTATGCCTTAGCTCAAGTGTTTCATTTCTGTATTTTTGGTTAACAGGCTAATCGAGGAGAGCTCTTCAGTGATGGAGGCAGCATACAGCTGCCATTGGTACGACGGTTCCGAAGAGGCAAAAACTTTTGTGCAAATTGTTTGTCAACAATGTCAAAAAGCCATGTCCATATCGGGGGCTAAATTTTTTACCATATCTCTCGACTTATTTGATTCCGGTCCGCCATCAAATACTGGGTCGAAAGGCATAAGCATGTCGTAAGACTAGTCACCGCAATCGGGGACAGTTATGGGGTAGCTTTACTATTGCATATGTTAACATCTACAGTAATGCTAACTCTTCTTGCGTATCAGGCCACAAAGATTACTGGCGTTACCCCGTATGCAGCGTCTGTACTGGGTTACCTGGTCTATGCCTTAGCTCAAGTGTTTCATTTCTGTATTTTTCGTAACAGGCTAATCGAGGAGAGCTCTTCAGTGATGGAGGCAGCATACAGCTGCCATTGGTACGA | [M](http://web.expasy.org/cgi-bin/translate/dna_sequences?/work/expasy/tmp/http/seqdna.13574,2,23) P N I R L [M](http://web.expasy.org/cgi-bin/translate/dna_sequences?/work/expasy/tmp/http/seqdna.13574,2,29) Q A S G H F L F N Y Y D N [M](http://web.expasy.org/cgi-bin/translate/dna_sequences?/work/expasy/tmp/http/seqdna.13574,2,43) G G [M](http://web.expasy.org/cgi-bin/translate/dna_sequences?/work/expasy/tmp/http/seqdna.13574,2,46) [M](http://web.expasy.org/cgi-bin/translate/dna_sequences?/work/expasy/tmp/http/seqdna.13574,2,47) H L I R V G Y C W T L L I L L L F Q F G G A F G N L I V E A D D V N D L A A N T I T V L F F T H C I T K Y V Y F A V R S K L F Y R T F G I W N Q S N S H P L F V E S N N R Y H A V A L K K [M](http://web.expasy.org/cgi-bin/translate/dna_sequences?/work/expasy/tmp/http/seqdna.13574,2,141) R I L L S A V V A F T V C S A I A W T T L T F F D E S A R E R P D P E N E N I T V T T Q L P K L L V K S W L P Y N A [M](http://web.expasy.org/cgi-bin/translate/dna_sequences?/work/expasy/tmp/http/seqdna.13574,2,200) S G [M](http://web.expasy.org/cgi-bin/translate/dna_sequences?/work/expasy/tmp/http/seqdna.13574,2,203) K H S L T F G F Q V Y Y V F I S [M](http://web.expasy.org/cgi-bin/translate/dna_sequences?/work/expasy/tmp/http/seqdna.13574,2,220) I H A N L [M](http://web.expasy.org/cgi-bin/translate/dna_sequences?/work/expasy/tmp/http/seqdna.13574,2,226) D V L L C S W L I F A C E Q L [M](http://web.expasy.org/cgi-bin/translate/dna_sequences?/work/expasy/tmp/http/seqdna.13574,2,242) H L K E I L K P L [M](http://web.expasy.org/cgi-bin/translate/dna_sequences?/work/expasy/tmp/http/seqdna.13574,2,252) E L S A T L D T Y V P K T A D L F R A P S T K S Q D K L I E N D Y N E K IA [M](http://web.expasy.org/cgi-bin/translate/dna_sequences?/work/expasy/tmp/http/seqdna.13574,2,291) I FRSAIKYWVERHKHVVRLVTAIGDSYGVALLLHMLTSTVMLTLLAYQATKITGVTPYAASVLGYLVYALAQVFHFCIFRNRLIEESSSVMEAAYSCHWY |
| **15** | G3QO8C008JYTYH ***Ap*OR64** | AGATTGAATAAGAAAAACGTGCAATCGAAACGAAAGTTGGTAGTGTTAACAATGTAAGTTTACCACCCGTGAAAATAACGGGTTTTCCCAATCGACACATGGAAAAGACCATTTTCATTTTGAAGGATTTAGAAGCGCATAGCCAATCTCCTTCAAACAGGGCATCAGATGTCGCCAAACTCATTTGTGTGAGTTTATTTCCATACCAACAATATTGAAAAATTTGCCAAGTTACAGCCAGAAGGTATGCCAATTCTGCAAAGAATTTCCCACTCGTTATTGGTACCTCAGAAACCACATATAGACCTGCACAAAGAACCGTCACAGATTGTAGAATTTGTAAAAGGGCTATAACATTAAAGGTGTCTTCGATAGTCTCGCACATCCT | [M](http://web.expasy.org/cgi-bin/translate/dna_sequences?/work/expasy/tmp/http/seqdna.4201,4,2) C E T I E D T F N V I A L L Q I L Q S V T V L C A G L Y V V S E V P I T S G K F F A E L A Y L L A V T W Q I F Q Y C W Y G N K L T Q [M](http://web.expasy.org/cgi-bin/translate/dna_sequences?/work/expasy/tmp/http/seqdna.4201,4,69) S L A T S D A L F E G D W L C A S K S F K [M](http://web.expasy.org/cgi-bin/translate/dna_sequences?/work/expasy/tmp/http/seqdna.4201,4,91) K [M](http://web.expasy.org/cgi-bin/translate/dna_sequences?/work/expasy/tmp/http/seqdna.4201,4,93) V F S [M](http://web.expasy.org/cgi-bin/translate/dna_sequences?/work/expasy/tmp/http/seqdna.4201,4,97) C R L G K P V I F T G G K L T L L T L P T F V S I A R F S Y S I |
| **16** | isotig00970**_*Ap*CSP4** | AATGAATAGTTTTGTATTTATTTAAGAAAATAAATGACAATTGTCCATCCCCCCCCCCTT  GTCATCGCAACTATTGAATCTAAAATTTTGAAAAAATTCAGAAAATAGTTCTTTACTTAT  AATTACCTACAGGAAAATTAATAAGAAGCCTATGGTCTTATCATAATGTAGTAAATCATT  CAACAAAACAATGTTGTGCGTACAAATCTAAATTTGCAGTTTTAATGTATGAGAGAACAT  GTTTTGTTTCAACTACAATGTTAAGTTAGTTCTTAGGTTTAGGTTCCGTACATCCGAATA  AAAAACAGATCACTCGTGTATCTGTCTGTCCGTTTTATGTTTGTTTTTTTTGTCTGATAA  CACTAGCATAATTCGGCAAAATAACTTATTTTATTTTTTAATAATGTTGGAGCTAAATTA  CTGGACATAATGGTGGGCCATTCTTTACTTCCTTATCCTGAAAATTATAAGGTATTTTAT  TATACTGCAGTTAACAGTTGGCGGGGCAATTAAAATGAACGAACTCTATATCCATTTAGT  TGACCTGCCTGTATTAATATACATTGTTTGCAATTTTACAATACAATTCTATTTTTATAA  ATATCTCCACAATCTCTACAGCCCATCTAATTACATTTTTATTTGTTCTGAATTAATGTC  ATATTAGCATAATTTTGATTTAAACTACTTATAACATATTAGTATAACATAATCTATTTA  GTTTGAATTGCTTAAGAATTTTGTTTCTTAAGTTCTCCTAACAAAAACGCCTCATATTCC  CCAGAGGGGTCATATTTTGATAATAGTAATTTCCATTCGTCTGGTTTCTTCTCGATTAAG  AACTTGGAAGCTTTAAGCACCACTTTTCTTTGAACATCTGTACAATCTCCACATTTTGAT  TTAATAAACACTGGTATATCCTTTTTAAAATCACGAGCTTCCGGTGAACAAGGGCCTTTA  TCCAAGAAGCAGTTAATGTAGTTCTTAAGGATTCTTTCGTTTGAAAGAATCGAATCTAGG  TCTAGCGCTTCATACCTTTTAGAATAGTCGGATGCATCTCCCAATGAGTTAAATGTAAAA  ATAGTAAATACCAGAAACAAACTAACAATTGCGCTCCTCATAATGGATGAATTCTACTGG  AAAACTAGGCCC | **M** R S A I V S L F L V F T I F T F N S L G D A S D Y S K R Y E A L D L D S I L S N E R I L K N Y I N C F L D K G P C S P E A R D F K K D I P V F I K S K C G D C T D V Q R K V V L K A S K F L I E K K P D E W K L L L S K Y D P S G E Y E A F L L G E L K K Q N S |
| **17** | isotig01662**_*Ap*CSP10** | ACACTTCAAAGTTGTTAAATATATTTAACAGCAGATATTGTTTACAAATTAGTTGCTTTT  AACTACTGTAACTACTAATTTTATTGAAGAAATTGTATGTCATTTACATTTTAATATTAA  ATGGCAAGAAAACTCACTTTTTTCATCTCAATCTATGAAGAACAAGTACTACGTTTACGT  TTTTTTTAAACGTTGCGCAAAAAAATTCTGCATGATGCAAATGCTTAACAGTGTTTGGTA  TTACTAATTTTCGAGTTTTAGATGGAAATAGTTTCAGCATTTGACAAATTACAATTTAAT  TCCTTCCTTTTCTAGTTCATCTTTGTAATCTTTAACATATTGACCTTCGGGATCATACTT  CTTTTCCAATTTTTTCCACCAATCCTGTTTATTCTTAATCAAGAAATTAACCACTTTTTT  GCTACCTTGCTTTTGCTTATCACTGCAACCGTCACACTTGTGTTTAAGGGCATCGGGTAG  AACCTTTTTTAATTCAGCTCCATCAGGAGTACATTTTCCTTCCTCCATCAAGCATTTAAC  ATAATTTCCCAGGAGGCGATCGCTCTTCAGGATCTCGTCCAGGTCGATGTTGTCGTATTT  GGTCGTGTATTTGGTCGCAGACATCGCATAGGCGATGCATGACAAGACAACAGCAAAAAG  AAAACAACAGACGCCTTCATTTTCACTTATAACACTTGTGCAAACTGCCC | **M** S A T K Y T T K Y D N I D L D E I L K S D R L L G N Y V K C L **M** E E G K C T P D G A E L K K V L P D A L K H K C D G C S D K Q K Q G S K K V V N F L I K N K Q D W W K K L E K K Y D P E G Q Y V K D Y K D E L E K E G I K L |
| **18** | isotig02537**_*Ap*CSP3** | AGTCGTATCGTCTGTACCACAGAATGGAAAATACACCATCAAGTACGACAATGTCGACTT  GGATCAAATCCTCAGCAACCAAAGGCTACTCGAAAATTACTACAACTGCCTTATGGACAA  AGGAAAGTGTACTCCCGATGGACAAGAACTGAAAAAAATCTTCCTGATGCCTTGAACACC  AAGTGCTCCAAATGTAGCGAAAAACAAAAGGAAGGAACCCACAAAGTTGTTGAATATTTG  ATCAAGAACAAAAACGATTGGTGGAAGAATTTGGAAAGTAAATACGATCCCAGTGGCAAC  TACCGAAGAGACTATGGACCTGAACTAGCCCAAAGAGGCATCAAAATCTAAACGTTCCTA  TGAAAAAGTTGTTACCTACTAATAATGTATTGTCAAAAAAGTCATGTTTATTGTTTCATT  GGTAAACTTAATTGAAATTGTGCAATAAAGCTTTTTAAAGA | S R I V C T T E W K I H H Q V R Q C R L G S N P Q Q P K A T R K L L Q L P Y G Q R K V Y S R W T R T E K N L P D A L N T K C S K C S E K Q K E G T H K V V E Y L I K N K N D W W K N L E S K Y D P S G N Y R R D Y G P E L A Q R G I K I |
| **19** | G3QO8C008JTBQU_***Ap*CSP1** | GATAGTGTGCTTTGAAACCGAAAGAAGAAAACAAGTTTACTAAAAAAATACAAAAATAGAAAAGTTTTGCAATTGTTACGCTTTTGGCCGTTGCCATGGCAGTGGTTTATGCCGCTCCCG  ATGGCGTTAAGTTCACCACCAAGTACGACAACATTGACCTGGATGAAATTCTACCAAAAC  GAGAGACTTTTCAACAACTACTTCAAGCGTCTAACCGGCGAGGGAAATGCACCCCTGACG  GCGAAGAACTCAAAAAGGCATTCCTGAGGCCTTAAAGAACAAGTGTGGAGGATGCAGACG | KSFAIVTLLAVAMAVVYAAPDGVKFTTKYDNIDLDEILPKRETFQQLLQASNRRGKCTPDGEELKKAFLRP |
| **20** | 000793_EAB-5_isotig02106_*Ap*Igr | AAGAAATAATACAGAACCTCCAAAGTCAACTTCTTTTCTTTGAAGGGCTCCTGTCATACC  ATCAAAGGAACCGTTTGCAAGTTTAAAACCCCAAGAATCGGTTAAACTAAAGTTTAATGA  GAAATTGTAAATTTCTTTGCAGTGATTCATAAGTACACTGTGAAACTTGGTACGAGAATC  GACCCTATAATTATTTTCTTTCAAAATGTATTCTTCTAATTCACCCTCGAATTTATTAAC  CAACACTGCAATTGTTTTAAATACAACGCCACTCATATTTTTTCGGTTCCAATATTTATT  GTAATTGTTTGATTTTAAATTAAATGAAAAATTATTCGATTTTTTACTAGCACGTTGTAA  TTTAATTTCCCCCCTTTTTGTAAGCGGCATTGTACACATCTATCAACGTCACATTTACAT  TAACTCCGTTTCCGTATTCGCATTCCGCCACTAACAGATTTGAATTTATATTTGTATTTA  CATTAGAAAAAACTTTTTTAATAAATTTCTTTGTGATTATCAACCAGTGATAGGAATCGT  TAAAAAGTTCAAATTCGCCAGC | [M](http://web.expasy.org/cgi-bin/translate/dna_sequences?/work/expasy/tmp/http/seqdna.4829,5,55) C T [M](http://web.expasy.org/cgi-bin/translate/dna_sequences?/work/expasy/tmp/http/seqdna.4829,5,58) P L T K R G E I K L Q R A S K K S N N F S F N L K S N N Y N K Y W N R K N [M](http://web.expasy.org/cgi-bin/translate/dna_sequences?/work/expasy/tmp/http/seqdna.4829,5,96) S G V V F K T I A V L V N K F E G E L E E Y I L K E N N Y R V D S R T K F H S V L [M](http://web.expasy.org/cgi-bin/translate/dna_sequences?/work/expasy/tmp/http/seqdna.4829,5,138) N H C K E I Y N F S L N F S L T D S W G F K L A N G S F D G [M](http://web.expasy.org/cgi-bin/translate/dna_sequences?/work/expasy/tmp/http/seqdna.4829,5,169) T G A L Q R K E V D F G G S V L F L |
| **21** | 000793_EAB-5_G3QO8C008I50AS *Ap*Igr | TTATCGTGTGTGTCTGCAGGATGCGTCAAAACCCATTCCCATCCGAATACCATTTGGAGA  GGTATAAATGCCAATAATATGAGAAGTCCACAGGCAATCCATACATCCGTACCGAAAGTT  AAAGTATATAAATTGTACATCGTTGATAATGGCGGTTCCTTCAAAAGAAAGCCACACTAG  CAGGAGTTGTAGATCCTATATACTCCACTATGTGCAGGCGTGTTGTGTCACCCGCTTATT  ACTGTACCTGAGATGTCTGTCAAACCGGAAATAAATCTTGCATGAGTGGATCAATTTCAC  TTTCTGTTTCCGTGATAATCCCCACGCATAATGCATACGATAGGCTATCGATGCA | [D](http://web.expasy.org/cgi-bin/translate/dna_sequences?/work/expasy/tmp/http/seqdna.7676,5,54) L Q L L L V W L S F E G T A I I N D V Q F I Y F N F R Y G C [M](http://web.expasy.org/cgi-bin/translate/dna_sequences?/work/expasy/tmp/http/seqdna.7676,5,85) D C L W T S H I I G I Y T S P N G I R [M](http://web.expasy.org/cgi-bin/translate/dna_sequences?/work/expasy/tmp/http/seqdna.7676,5,105) G [M](http://web.expasy.org/cgi-bin/translate/dna_sequences?/work/expasy/tmp/http/seqdna.7676,5,107) G F D A S C R H T R |
| **22** | 000793_EAB-5_G3QO8C008JKL7H *Ap*Igr | ACTTTTTTGTTTCCTTTCTTCGATGGCGACTTTCCGTGATTTCCAGACGAATTCGCACAC  TGCAATGACGCAAGCTACCCCCATGCCGCCCATAAGGACAACAAACACCCCACCGATATT  TGCAAGACCCAAAGCGTTCGCTGTACTGCTAGTTTTCGTAGTTTCATCATTGCAAGCACC  TCCACCCCGTTTCTCCTTCCACCATCTCGTCTTCAGTACGTGCTAATTTTTCCTTCTTCT  TGGAGCTTGAGGATGGCTCCACTTATTGCAGTTCGAAACGGTGAATTTGGAGCATTGCG | [F](http://web.expasy.org/cgi-bin/translate/dna_sequences?/work/expasy/tmp/http/seqdna.12487,1,14) P D E F A H C N D A S Y P H A A H K D N K H P T D I C K T Q S V R C T A S F R S F I I A S T S T P F L L P P S R L Q Y V L I F P S S W S L R [M](http://web.expasy.org/cgi-bin/translate/dna_sequences?/work/expasy/tmp/http/seqdna.12487,1,85) A P L I A V R N G E F G A L |
| **23** | 000793_EAB-5_G3QO8C008JH3EB *Ap*Igr | GCATCCTGCAGACACACACGATAATCGATGCCAGATGATAACAAGATCTAACCTTTCCGA  CACGACATTGTTTCAAGTCGAGATGATATGTCAACAGGGCACAAACGTTGTTCCAAAAAG  TATATCCGGAAGGATCTTGGTTCTTATTTTGCTTATAGCTTTTATGTTCTTGTACTAC | [V](http://web.expasy.org/cgi-bin/translate/dna_sequences?/work/expasy/tmp/http/seqdna.12838,4,1) V Q E H K S Y K Q N K N Q D P S G Y T F W N N V C A L L T Y H L D L K Q C R V G K V R S C Y H L A S I I V C V C R [M](http://web.expasy.org/cgi-bin/translate/dna_sequences?/work/expasy/tmp/http/seqdna.12838,4,59) |
| **24** | 000793_EAB-5_G3QO8C008JFTC4 *Ap*Igr | GCATCCTGCAGACACACACGATAATCGATGCCAGATGATAACAAGATCTAACCTTTCCGA  CACGACATTGTTTCAAGTCGAGATGATATGTCAACAGGGCACAAACGTTGTTCCAAAAAG  TATATCCGGAAGGATCTTGGTTCTTATTTTGCTTATAGCTTTTATGTTCTTGTACACTTC  TTTTTCCGGTAATATTGTGGCATTTATTACG | [R](http://web.expasy.org/cgi-bin/translate/dna_sequences?/work/expasy/tmp/http/seqdna.14156,4,1) N K C H N I T G K R S V Q E H K S Y K Q N K N Q D P S G Y T F W N N V C A L L T Y H L D L K Q C R V G K V R S C Y H L A S I I V C V C R [M](http://web.expasy.org/cgi-bin/translate/dna_sequences?/work/expasy/tmp/http/seqdna.14156,4,70) |
| **25** | 000793_EAB-5_G3QO8C008JMA0O *Ap*Igr | TCAAAAAATTGGCCACTGGCATAACAAAACTTCTAACAAAATAAAGTATAAGTACGGAAAATTTCATAGAAGACGAAATATGTCGGAAGTAAAATTTAAAACAGCGCTAGTGATCACATTAAATGGTACCGTTGATGTTGATAAATATGCACTTGATGAAACCAGTCGTCAAAACAATACTCACAGTAGATACCACAGTGTCGCAATGAGAATCTGCAGGGATTACTATAATTTTTCAATCGAATTGATGGGCACAAACTCATGGGGTTACGAAAAGTCTAATGGCACATTTGATGGATTAGTAG | [Q](http://web.expasy.org/cgi-bin/translate/dna_sequences?/work/expasy/tmp/http/seqdna.15198,2,1) K I G H W H N K T S N K I K Y K Y G K F H R R R N [M](http://web.expasy.org/cgi-bin/translate/dna_sequences?/work/expasy/tmp/http/seqdna.15198,2,27) S E V K F K T A L V I T L N G T V D V D K Y A L D E T S R Q N N T H S R Y H S V A [M](http://web.expasy.org/cgi-bin/translate/dna_sequences?/work/expasy/tmp/http/seqdna.15198,2,69) R I C R D Y Y N F S I E L [M](http://web.expasy.org/cgi-bin/translate/dna_sequences?/work/expasy/tmp/http/seqdna.15198,2,83) G T N S W G Y E K S N G T F D G L V |
| **26** | 000793_EAB-5_G3QO8C008JRMP2 *ApG*r | ACAAGAGGAATATATTTATATAGGAGATGCACATGACGCAGAGTTCCTTGCATTGACCGA  CTGTGACTTAAAAGCAATCGGAAGTGAGTTTTCTAAAAGGCCGCATGGCTTTGCTCTTCA  AAAGGATTCACCATTGAAAGAAATTTTCGATCAAGCGTTTATCGAACTCGTGAGAAATCA  CGAATATGATCAATTAAAAGAGAAGTGGTGGGAACAAAAACCCAAACAAGCGATATGTAAATAAAGAACAAGAAGGAATTGATTTAAGCC | [Q](http://web.expasy.org/cgi-bin/translate/dna_sequences?/work/expasy/tmp/http/seqdna.16118,2,1) E E Y I Y I G D A H D A E F L A L T D C D L K A I G S E F S K R P H G F A L Q K D S P L K E I F D Q A F I E L V R N H E Y D Q L K E K W W E Q K P K Q A I C K |
| **27** | 000793_EAB-5_G3QO8C008JI640 *ApG*r | GTTCACCATGAGTAATACTGAAGGGGTTGAAAGGGTCGTTAAGGGCAAAGGCAGTTACGCCTTCTTGATGGAGTCGACGACAATCGAGTATGTTATCGAGAGAAACTGCGAACTGACACAAATCGGAGGACTACTCGACTCGAAAGGGTACGGGATCGCAATGCCTCCAAATTCACCGTTTCGAACTGCAATAAGTGGAGCCATCCTCAAGCTCCAAGAAGAAGGAAAATTGCACGTACTGAAGACGAGATGGTGGAAGGAGAAACGGGGT | [F](http://web.expasy.org/cgi-bin/translate/dna_sequences?/work/expasy/tmp/http/seqdna.16509,2,1) T [M](http://web.expasy.org/cgi-bin/translate/dna_sequences?/work/expasy/tmp/http/seqdna.16509,2,3) S N T E G V E R V V K G K G S Y A F L [M](http://web.expasy.org/cgi-bin/translate/dna_sequences?/work/expasy/tmp/http/seqdna.16509,2,23) E S T T I E Y V I E R N C E L T Q I G G L L D S K G Y G I A [M](http://web.expasy.org/cgi-bin/translate/dna_sequences?/work/expasy/tmp/http/seqdna.16509,2,54) P P N S P F R T A I S G A I L K L Q E E G K L H V L K T R W W K E K R G |
| **28** | ***Ap*SNMP1** | ATTAAACCCTTGTTTTATTATTATTATTTCTTTTGTTTCATCTACAGGTACATAAGTACA  CATAACACTGCAAAATTTGTACAAGAAGTTTTCGATGTTTTACTTTCTCTAACTCTTTTG  CCTATTCTGCTTAAGAAAATCTAACAACATGTGATCAGTATCTACTAATTCATAAATCAG  GTACTTCAATAATCAGTATTTGTTATTGGATCCGTTCACGTAAGTGTTCAGGGAATCAAT  AGTATTTATTACAGAACTCCTGCCTTTCGACTCGTCATTTTTTACAGGTGTGACAAGGAT  ATTTTTCGAATTTTTGAAATGCATTAAACCGGCAATTGCACCTGTAACTGCAGACACTAC  AAATAGTATCCACTTTGCCGTAAAAACAATTTGAATCATCTTGAATGCAGATTTAATCTG  ATCAACATATTCGTCACCCAAATCTGCACCCTCTTCTACCCAAAACACAGGCAATATTGT  AGTGGGCAAATTTTTCATAAAACTAATTTTCGGTATCGGTTCTAAGGGCATACTGAACTG  CAGCCTTTTTCTTGCTCCTAGGGGTGTTCCTGTCATAGGTTCAAAATACATATAAATTCC  ATGTTCCTCTTCGTTTGGATGTAATCCACGAACGCCATGAACGTAACTTTCATCAGACGC  ATAGAAATGCGGCAAGGATGCGTATAAGGGAGCACCAACGCACTTTGTTATTTCCATTAA  TCCTTTCTTTAAGCACTTATTAGGTTCAGGACAGTAACATTTCAAACTATCATCCGCAGA  CATATCCCCTAAAGTTGCAGTGTATTTGTTCACTTTGACTTCTTTAACAAACGTTTCTTT  TTCGAAAACTGCGGCTAGTGATCTACAAATATCGGGGGAAAATGCGACAATACCTTCTTC  CTTTTGAAGAAGGGGTGGAAAAATTGTCGAATCAGTACCGTGATACTCATTACACTCCTT  TGTGGGCCAAACATTCATATTTGGTTTATTGTCAAACTCAACAACCCTTCCAATATCCTT  ATAATTTTTTAATCCCCGCTTAACCGTGAATGGCCTCTCATCAACCGTACCGTTTTTATC  TCCAAAAAAGGACAATTTAAATATCTTGTCGCTGATGTATATTACATTTTTTGCTTCCGA  TTTTATCTGAGCGCAAGCCGCCTTCCCAGCAAAATCCGTCACATTGCAGTAAACAGTCAA  ACCTTCGAAGAGGATTTCGTTCGTTGTAGCGGTAATGAAGGGCGATGGTGGATTATTAAA  GATTGAATTTAATGCTTTCGAAACTAGCGATAACATTGCTGGTTTGTCCCTAGCAACAGC  AACAGCTATGCCCAGAATTAACGGATGTGGAATAGTAACCATTTCATCCCCTGTTAATGG  TGCGGATTTGGCTTTATTAAAAATCCACGTCATTTTTCCCTTATATGTTAGAGTGTCATT  AACTTCATTGTCTATAGGATCGACTTTCTCTTTCCACAAATCGTAACAGTAGGGTCCAAT  TTCATCTAACACTGGACTCTCCCCATTAAGTACGGCTTCGGCATTAGTAATATTAAACAT  ATAAACTTTAAAGTCCAAAGCAAACGGGGTCTTCATGTACATTTTTCTTATTTCACTACC  AGGTTTTAGTGCTACTTGTTGCTTTCACTTTACTTTTCACCATTTTCGGAAACATTATAA  ATCCAAAAATAACAACAACAACAAATACTATTCCAGAGG | [M](http://web.expasy.org/cgi-bin/translate/dna_sequences?/work/expasy/tmp/http/seqdna.15201,4,40) Y [M](http://web.expasy.org/cgi-bin/translate/dna_sequences?/work/expasy/tmp/http/seqdna.15201,4,42) K T P F A L D F K V Y [M](http://web.expasy.org/cgi-bin/translate/dna_sequences?/work/expasy/tmp/http/seqdna.15201,4,54) F N I T N A E A V L N G E S P V L D E I G P Y C Y D L W K E K V D P I D N E V N D T L T Y K G K [M](http://web.expasy.org/cgi-bin/translate/dna_sequences?/work/expasy/tmp/http/seqdna.15201,4,103) T W I F N K A K S A P L T G D E [M](http://web.expasy.org/cgi-bin/translate/dna_sequences?/work/expasy/tmp/http/seqdna.15201,4,120) V T I P H P L I L G I A V A V A R D K P A [M](http://web.expasy.org/cgi-bin/translate/dna_sequences?/work/expasy/tmp/http/seqdna.15201,4,142) L S L V S K A L N S I F N N P P S P F I T A T T N E I L F E G L T V Y C N V T D F A G K A A C A Q I K S E A K N V I Y I S D K I F K L S F F G D K N G T V D E R P F T V K R G L K N Y K D I G R V V E F D N K P N [M](http://web.expasy.org/cgi-bin/translate/dna_sequences?/work/expasy/tmp/http/seqdna.15201,4,248) N V W P T K E C N E Y H G T D S T I F P P L L Q K E E G I V A F S P D I C R S L A A V F E K E T F V K E V K V N K Y T A T L G D [M](http://web.expasy.org/cgi-bin/translate/dna_sequences?/work/expasy/tmp/http/seqdna.15201,4,313) S A D D S L K C Y C P E P N K C L K K G L [M](http://web.expasy.org/cgi-bin/translate/dna_sequences?/work/expasy/tmp/http/seqdna.15201,4,335) E I T K C V G A P L Y A S L P H F Y A S D E S Y V H G V R G L H P N E E E H G I Y [M](http://web.expasy.org/cgi-bin/translate/dna_sequences?/work/expasy/tmp/http/seqdna.15201,4,377) Y F E P [M](http://web.expasy.org/cgi-bin/translate/dna_sequences?/work/expasy/tmp/http/seqdna.15201,4,382) T G T P L G A R K R L Q F S [M](http://web.expasy.org/cgi-bin/translate/dna_sequences?/work/expasy/tmp/http/seqdna.15201,4,397) P L E P I P K I S F [M](http://web.expasy.org/cgi-bin/translate/dna_sequences?/work/expasy/tmp/http/seqdna.15201,4,408) K N L P T T I L P V F W V E E G A D L G D E Y V D Q I K S A F K [M](http://web.expasy.org/cgi-bin/translate/dna_sequences?/work/expasy/tmp/http/seqdna.15201,4,441) I Q I V F T A K W I L F V V S A V T G A I A G L [M](http://web.expasy.org/cgi-bin/translate/dna_sequences?/work/expasy/tmp/http/seqdna.15201,4,466) H F K N S K N I L V T P V K N D E S K G R S S V I N T I D S L N T Y V N G S N N K Y |
