# Supplementary material for: Identification of Odor-Processing Genes in the Emerald Ash Borer, Agrilus planipennis
Source: PLoS One. 2013 Feb 12;8(2):e56555. doi: 10.1371/journal.pone.0056555 (PMC3570424; doi:10.1371/journal.pone.0056555)
Supplement: Table S9 — Detailed analysis of A. planipennis odorant binding proteins. (DOC) [file pone.0056555.s013.doc]

**Table S9:Homology modeling of *Agrilus planipennis* OBPs against experimentally-derived insect OBP structures**. Homology modeling was performed against the best PDB match (as determined by BLAST) using the SWISSMODEL workspace (Arnold et al. 2006). Model and target OBP were overlayed in SwissPDB viewer. Model alpha helices are displayed in green and target helices in red. Disulfide connections between cysteines predicted by the model are also included.

| **Sequence name** | **Amino acid sequence** | **Best PDB match ID** | **PDB acc. no.** | **Superposed structures** | **Predicted disulfide bonds** |
| --- | --- | --- | --- | --- | --- |
| ApOBP1 | ELAKMLHDNCQGETGVPEEMIENARKGDFADDDRFKCYLKCVMVQMAVMNDEGVVDPEAVVAVLPDELKDVLSGSIRACGGKVGKDQCENAWLTHKCYYEKEPEHYFLV | Chain B, Crystal Structure Of Odorant Binding Protein 1 From *Anopheles Gambiae* (Agamobp1) With Deet (N,N-Diethyl-Meta-Toluamide) And Peg | 3n7hB | 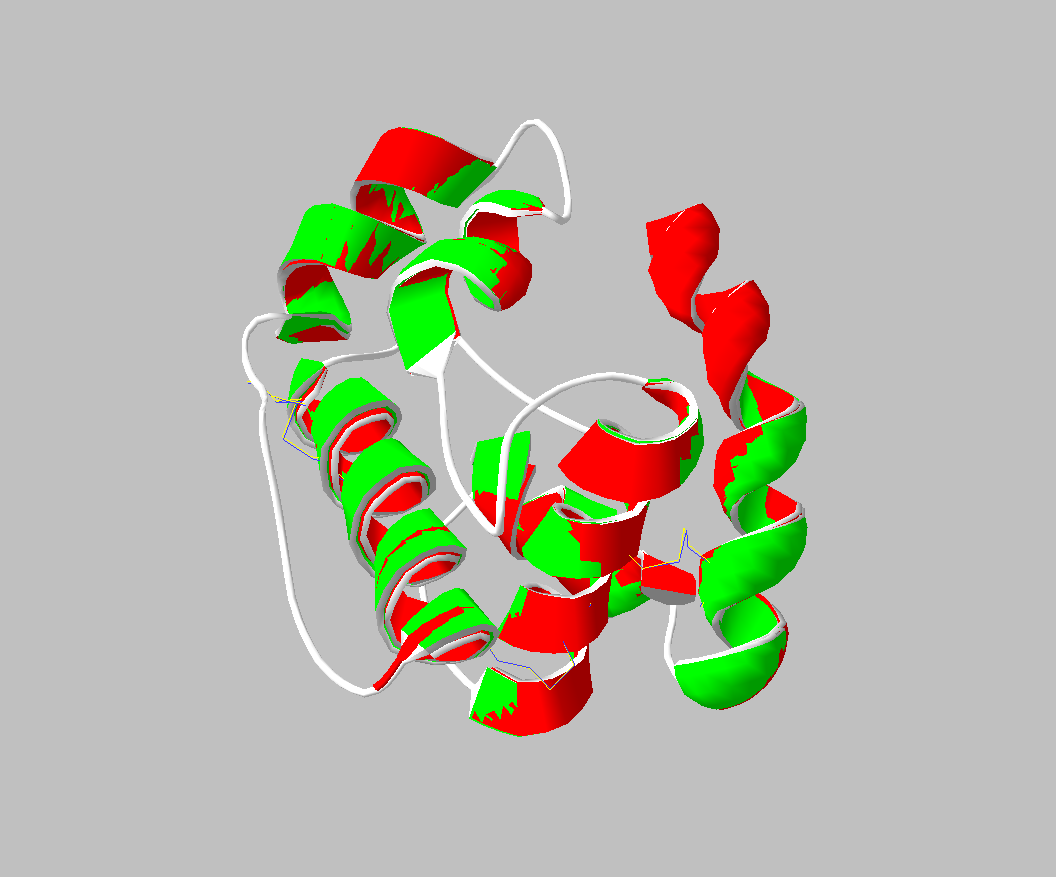 | C10-C41  C37-C88  C79-C97 |
| ApOBP3 | LENYPPPEVLEYLKPYHTICTEKIGVSDDEVKNYKIEDNSEKMMCYMRCLGLESKWLTPDNKLQIDYIMETRLDSIADLVKNIVDNCKDVPDGTHECEKAYNLHKCAAKIEPERWFLP | Crystal Structure of an Odorant-binding Protein From the Southern House Mosquito Complexed With an Oviposition Pheromone | 3OGNA | 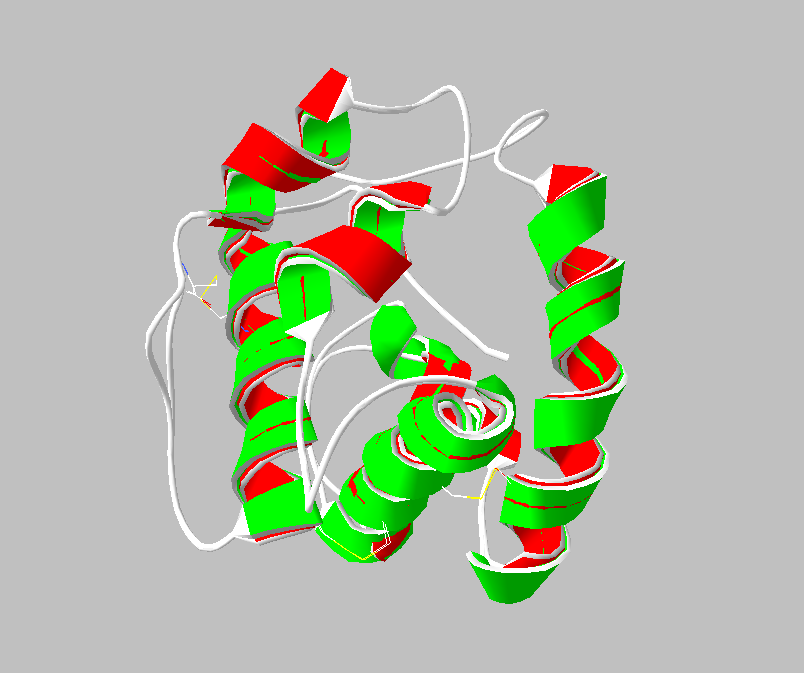 | C20-C49  C45-C97  C87-C106 |
| ApOBP4 | PMNEAQLQNAAKLIRNVCQPKLKISDKLIENIHNGDFAENEKVMCYLECVLRMGQLMKNGKFDEKAALSQISTLPPERQQATKDSIKKCADKGQDDDKCVAAFETAKCIYFDNPQNY | Chain A, Complex Of Drosophila Odorant Binding Protein Lush With Ethanol | 1OOFA | 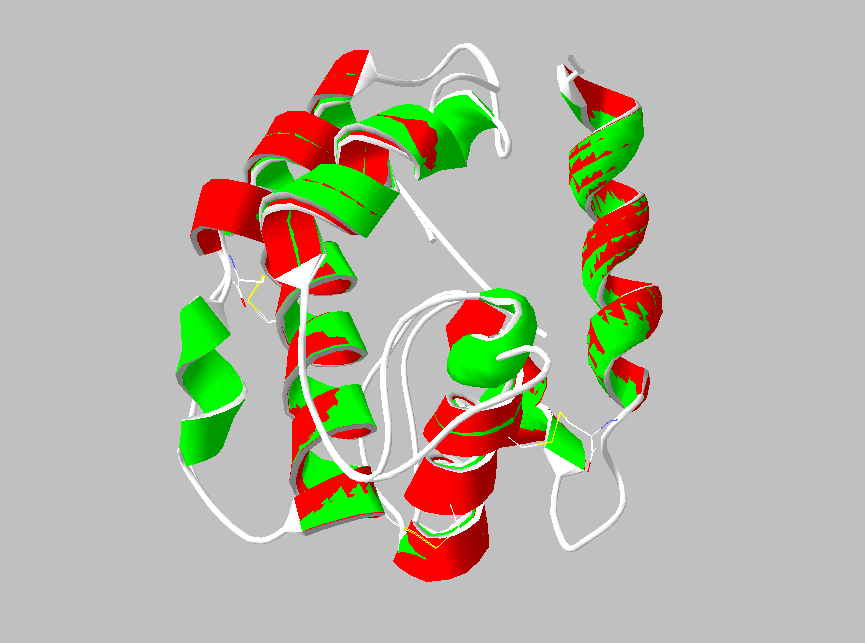 | C18-C49  C45-C99  C89-C108 |
| Isotig00242 | TPMNEAQLQNAAKLIRNVCQPKLKISDKLIENIHNGDFAENEKVMCYLECVLRMGQLMKNGKFDEKAALSQISTLPPERQQATKDSIKKCADKGQDDDKCVAAFETAKCIYFDNPQNYFLP | Chain A, Complex Of Drosophila Odorant Binding Protein Lush With Butanol | 1OOHA | 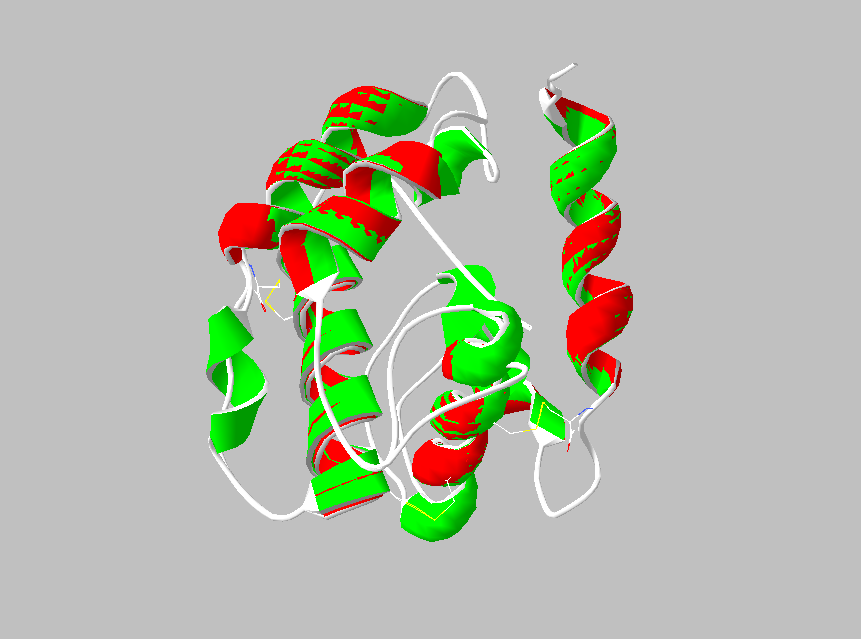 | C19-C50  C46-C100  C90-C109 |
| contig3672 | MTDEQKAKLKQHQDECLKSFPADKLLLEKARKGDLADNKTLKDYLYCVIEKSGFITPDGKIQTAVLETKLASVTAAENAKKLVAKCTSQKNLDKKESAEAIYKCVYNETKFSLI | Chain A, Thp12-Carrier Protein From Yellow Meal Worm | 1C3YA | 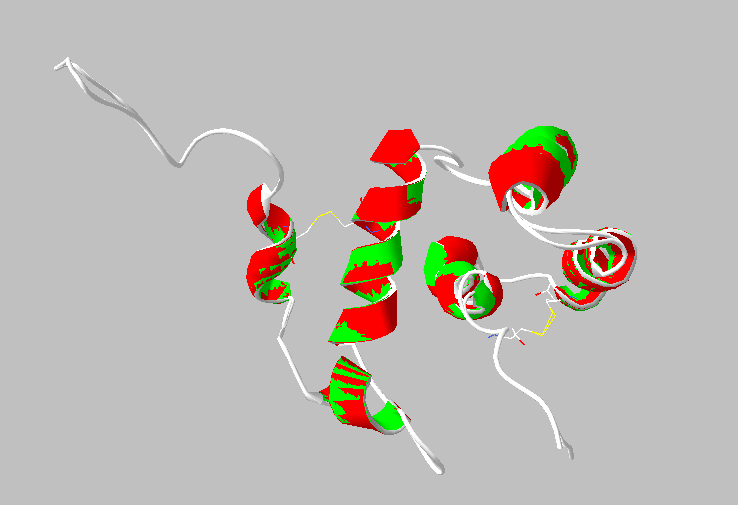 | C16-C47  C86-C104 |
| Isotig01620 | NSFSENRHHAPIFEVCCEFEPFMAAPEDEKMKECISEILGDPKSMNHSNPPTDQELEELAEKIACIEECTAKKYELLDSDGNIVLAKLLEVAQGKINGTFMENHIEEAAKKCIEDIEKEIPKESKCNPKPLFLSNCLFFRSLENCPADQVKDKAKCETMIDEIKNGKFPHHFDPPPPEN | Chain A, Crystal Structure Of A Novel Type Of Odorant Binding Protein From Anopheles Gambiae Belonging To The C+ Class | 3pm2A | 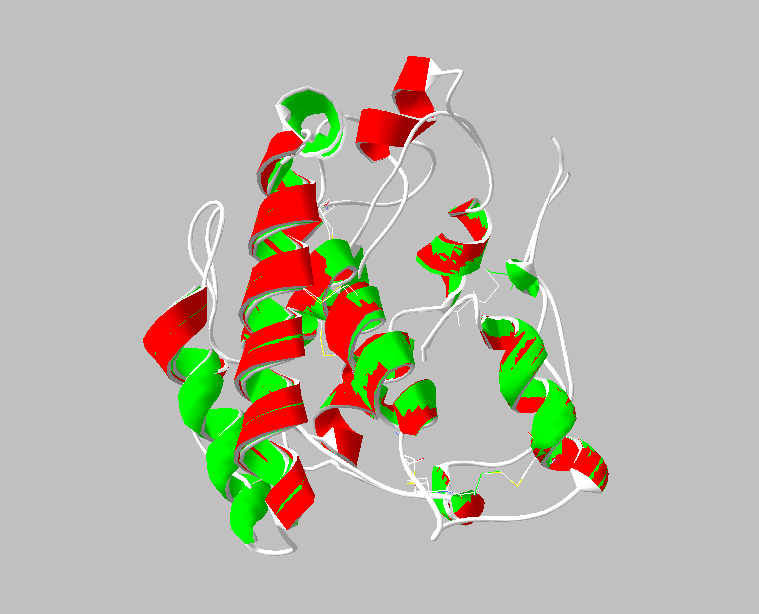 | C16-C156  C17-C145  C34-C69  C65-C126  C112-C136  (C5-C170 pair from model missing in target) |
| Isotig02192 | EEVNPFEEDIQHLLEMRKICEEKFPVSKETVESLKQGIYPSEADDPNVCENVLCVIKGRGFVDEDGNVMQKNFPEKMWNAMPENCKENRGSDICEKVKNFLPMSICRFSTAMGTNECRHLTRLDYHVLLNQL | Chain A, Complex Of Drosophila Odorant Binding Protein Lush With Butanol | 1OOHA | 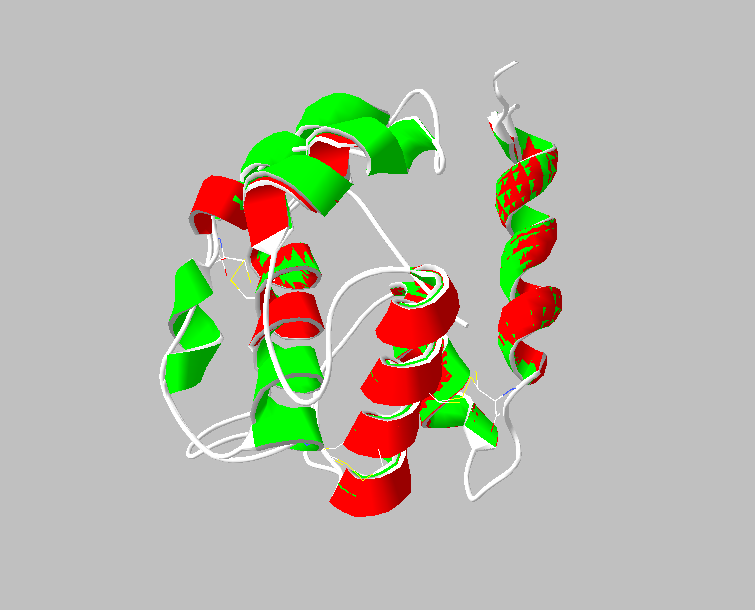 | C20-C54  C49-C94  C85-C106 |
| G3QO8C008JDOEQ_ singleton | SIVVLYLISTNADPGKSSTIEIREWENNLIKDELSCINSTGVSLSVIERTKVTLELPEDDPKYKEYLKCFYTKRGYQSDSGEVLYDNIKIMIHQFTNATEATRIIDLCKEMRGATA | Chain A, Thp12-Carrier Protein From Yellow Meal Worm | 1C3YA | QMEANscore4 too low | C36-C69  C108- |

Arnold K, Bordoli L, Kopp J, and Schwede T (2006). The SWISS-MODEL Workspace: A web-based environment for protein structure homology modelling. Bioinformatics 22:195-201.
