# Supplementary material for: Identification of Odor-Processing Genes in the Emerald Ash Borer, Agrilus planipennis
Source: PLoS One. 2013 Feb 12;8(2):e56555. doi: 10.1371/journal.pone.0056555 (PMC3570424; doi:10.1371/journal.pone.0056555)
Supplement: Table S10 — Primers used in the current study. (DOC) [file pone.0056555.s014.doc]

**Table S10**: Primers used in the current study.

| **Name of the gene with contig number** | **Forward primer (5’-3’)** | **Reverse primer (5’-3’)** |
| --- | --- | --- |
| ApCYP9 | GCAATCACATCAGTAGCA | TACCTCCAGCAAGAAGAT |
| ApCYP6 | TAATCCTGGCACGCATAC | CAGATATACGACCGATATTATTGG |
| ApOR83 | CGTTGTTGATTCTACTTCT | ATATACCGAATGTCCTGTA |
| ApOBP1 | CTTCAGGGTCCACTACTC | TTGCCGATGACGATAGAT |
| ApSNMP | GATACTCATTACACTCCTT | AATATACATCAGCGACAA |
| ApOBP2 | TCTCATCAATCATAGTCTCA | GAATCCAAGTGTAATCCAA |
| ApEF 1-α | CATTGAAACCTACGTTGTCGC | ACTGGAGTGCTTAAACCTGG |
